# Application of in vitro pulmonary models for hazard screening of silica particles

**DOI:** 10.1007/s00204-025-04100-5

**Published:** 2025-06-25

**Authors:** Nienke Ruijter, Hedwig Braakhuis, Alberto Katsumiti, Itziar Polanco Garriz, Marie Carriere, Ilaria Zanoni, Ana Candalija, Jessica Marshall, Jolanda Vermeulen, Flemming R. Cassee, Matthew Boyles

**Affiliations:** 1 National Institute for Public Health & the Environment (RIVM), Bilthoven, The Netherlands; 2 Present Address: TNO, Risk Analysis for Prevention, Innovation and Development, Utrecht, The Netherlands; 3 GAIKER Technology Centre, Basque Research and Technology Alliance (BRTA), Zamudio, Spain; 4 Univ. Grenoble-Alpes, CEA, CNRS, Grenoble INP, IRIG, SyMMES, CIBEST, Grenoble, France; 5 Institute of Science, Technology and Sustainability for Ceramics, CNR-ISSMC, National Research Council of Italy, Faenza, Italy; 6 Leitat Technological Centre, Barcelona, Spain; 7 Institute of Occupational Medicine (IOM), Edinburgh, UK; 8 Institute for Risk Assessment Sciences (IRAS), Utrecht University, Utrecht, The Netherlands; 9 Centre for Biomedicine and Global Health, School of Applied Sciences, Edinburgh Napier University, Sighthill Campus, Edinburgh, UK

**Keywords:** Nanomaterials, Hazard screening, Silica, In vitro, Inhalation

## Abstract

There is an increasing need for new approach methodologies (NAMs) for safety assessment of nanomaterials (NMs) in order to keep pace with innovation. In vitro assays are useful tools during pre-market hazard screening approaches of NMs to prioritize safe(r) candidate NMs and reduce the amount of regulatory testing required. For pre-regulatory hazard screening applications, it is crucial that in vitro assays have the capacity to distinguish between NMs based on their hazard potency and have the ability to provide accurate hazard rankings. In this paper, four types of silica particles (crystalline, pyrogenic, colloidal, and silane functionalized colloidal) were subjected to twenty-four in vitro assays to obtain hazard rankings using dose–response modelling. The assays were chosen for their relevance in the mechanism of action towards pulmonary inflammation upon inhalation of silica particles. The hazard rankings of silica particles were affected by cell type (alveolar or bronchial epithelial cells, macrophages), read-out method (cell viability, release of pro-inflammatory mediators, reactive oxygen species), and exposure method (submerged, air–liquid interface), complicating the assessment of the actual human hazard. Of particular note was an often muted in vitro response to the crystalline silica used in this study (DQ12), when in vivo data ranked this material as high hazard, due to the chronic and persistent in vivo inflammatory response to crystalline silica, highlighting an important functional discord between these models. However, the potency ranking of the silica particles to induce secretion of the pro-inflammatory mediator IL-1β by THP-1 cells differentiated to M0 macrophages as well as red blood cell haemolysis corresponded more closely to the hazard ranking based on data from rat inhalation studies. These assays should be further explored as indicators for human hazard potential of silica particles and other particles following a similar mechanism of action.

**Supplementary Information:**

The online version contains supplementary material available at 10.1007/s00204-025-04100-5.

## Introduction

There has been considerable growth in the use of new approach methodologies (NAMs) for safety assessment, which include in chemico, in vitro and in silico methods (Knight et al. 2021; Westmoreland et al. 2022). The development of these approaches is supported by the European Chemicals Agency (ECHA) (ECHA 2016), the US Environmental Protection Agency (EPA) (EPA 2018), and by the European Commission (EC), playing a pivotal role in their Chemicals Strategy for Sustainability (CSS) (EC 2020). However, the way towards regulatory uptake and acceptance of NAMs is long, as lengthy and costly assay standardization and validation processes and a general shift in mindset are required (Bas et al. 2021; Hoogstraaten et al. 2024). NAMs already play an important role in pre-regulatory hazard screening before they have been validated for regulatory testing, for example in a Safe and Sustainable by Design (SSbD) approach. SSbD is a concept which drives the design and development of safer and more sustainable chemicals and materials by incorporating safety and sustainability into the product design starting from the early stages of product development (Caldeira et al. 2022; OECD 2022; Soeteman-Hernandez et al. 2019). The SSbD approach is especially useful for nanomaterials (NMs) as they can be produced in an infinite amount of different nanoforms, and pre-regulatory hazard screening can help prioritization for regulatory hazard assessment (Soeteman-Hernandez et al. 2019). The SSbD concept would greatly benefit from the use of NAMs since in vivo hazard data are scarce during the early stages of product innovation (Abbate et al. 2024; Caldeira et al. 2022; Cassee et al. 2024).

The hazard assessment steps included in the SSbD framework presented by the Joint Research Centre (JRC) require elaborate hazard data (Caldeira et al. 2022), which proved challenging to fulfil even for chemicals already on the market (Caldeira et al. 2023). The NanoReg2 safe by design (SbD) approach for NMs makes use of hazard information ranging from very basic (in silico) information at the early stages of innovation (scoping and building of business case stages), to more complex hazard data in the later stages of innovation (development, testing, and validation stages) (Dekkers et al. 2020; Soeteman-Hernandez et al. 2019). In the earlier stages of product innovation, the emphasis of SSbD hazard screening should be on comparisons between candidate materials, or between an innovative material and the conventional material (Di Battista et al. 2024; Wohlleben et al. 2024). For the purpose of pre-regulatory screening of NMs in the context of SSbD it is therefore important that assays have the capacity to correctly rank NMs, prioritize safe alternatives, and flag NMs of concern (Ruijter et al. 2023).

In this work, we have used silica (SiO_2_) particles as a class of widely used materials with substantial information from both in vivo as well as in vitro studies, and a well-reported mechanism of action (MOA) leading to toxicity allowing for selection of suitable in vitro endpoints. Silica particles largely owe their toxicity to the organization of their surface silanol (Si-O-H) groups, which is in turn determined by their crystallinity, surface roughness, and synthesis conditions (Croissant et al. 2020; Pavan et al. 2020; Zhang et al. 2012). The crystalline quartz particle surface contains so-called nearly free silanol groups, which have a disorganized nature due to the fractured surface of the particle, and therefore possess membranolytic properties (Pavan et al. 2020). Inhalation exposure to quartz particles leads to silicosis and lung cancer, which is likely driven by a pro-inflammatory MOA (Borm et al. 2018; IARC 2012). The activation of the NLR family pyrin domain containing 3 (NLRP3) inflammasome is an important MOA of crystalline silica particles, leading to pro-inflammatory responses and cell death (Dostert et al. 2008; Hornung et al. 2008). The important relationship between the persistency of these adverse effects and a continued lung burden of inhaled particles has been reviewed recently (Poland et al. 2024). Non-resolving inflammation is considered critical for progression to lung fibrosis, with insolubility playing a key role in retention of inhaled crystalline silica (Arts et al. 2007). The surface of pyrogenic synthetic amorphous silica (SAS) contains reactive groups such as strained 2- and 3-membered siloxane rings due to their high temperature synthesis, which serve as reservoirs for reactive oxygen species (ROS) (Croissant et al. 2020). Pyrogenic SAS cause severe pulmonary inflammation upon inhalation, but this is partly resolved once the exposure is removed (Arts et al. 2007; ECHA 2019; Johnston et al. 2000; Merget et al. 2002; Reuzel et al. 1991; Weber et al. 2018). Colloidal silica particles do not have reactive surface groups because they are synthesized at low temperatures, and likely owe their (mild) toxicity to silanol groups alone (Zhang et al. 2012).

Pulmonary inflammation upon NM inhalation exposure is a commonly described adverse effect (Bomhard 2017; Braakhuis et al. 2014; Byrne & Baugh 2008). Pulmonary inflammation is challenging to capture in in vitro assays, but can be linked to factors induced by particle exposures which have been found responsible for ensuing inflammation, including generation of ROS, cell damage, changes in cell signalling, and pro-inflammatory cytokine release (Halappanavar et al. 2020; Inoue et al. 2021; Leikauf et al. 2020; Paur et al. 2011; Woo et al. 2023). These effects can be assessed in vitro using pulmonary cell types and/or immune cells either in a submerged exposure setting or at the air–liquid-interface (ALI) (Halappanavar et al. 2021; Paur et al. 2011), potentially serving as indicators of pulmonary inflammation.

The performance of several in vitro models was reviewed by Di Ianni et al. (2022) and in general demonstrated a good correlation between in vitro endpoints and pulmonary toxicity induced by NMs in vivo (using a comparison to a range of acute, sub-acute and sub-chronic studies). The complex models (such as ALI) did not necessarily outperform simple models (Di Ianni et al. 2022). Examples of promising cell models and read-outs identified in Di Ianni et al. (2022) were cytotoxicity of THP-1 cells (Pal et al. 2015), the in vitro NR8383 alveolar macrophage assay (Wiemann et al. 2016), IL-8 secretion by A549 cells (Di Ianni et al. 2021a, 2021b), cytotoxicity of a rat lung epithelial cell line (Han et al. 2012), and the haemolysis assay (Cho et al. 2013), but correlations differed per type of NM. In vitro models that include macrophages were previously shown to be useful for predicting pro-inflammatory responses for CuO NMs (Hufnagel et al. 2021), and carbon nanotubes (Clift et al. 2014). The use of combinations of multiple relevant cell types was shown crucial to avoid false negatives during in vitro screening of NMs in general (Xia et al. 2013). Acute pulmonary inflammation upon inhalation of quartz particles was shown by McLean et al. (2023) to correlate well with cytokine release (IL-6 and IL-1β) induced by quartz in simple submerged in vitro systems. Although these results are promising, there is more work needed to ensure there is adequate and specific knowledge to allow appropriate selection of in vitro assays and cell models for hazard screening and to have confidence in their performance.

In this study we tested four silica particles with distinct toxicity profiles, namely crystalline DQ12, pyrogenic NM-203, colloidal Silica-Std, and Silica-Silane, a colloidal silica with silane surface functionalization, shielding the surface of the particle from surrounding biomolecules (Ag Seleci et al. 2022; Book et al. 2019). We evaluated a wide range of in vitro toxicity assays and cell types for their capacity to distinguish between in vitro hazard potency of these silica particles. To this end, in vitro hazard potency rankings were established using assays targeting secretion of pro-inflammatory markers, ROS production, membranolysis, and cell viability, which are expected to be adequate indicators of hazard. The obtained in vitro hazard potency rankings were then compared to a hazard ranking based on rodent inhalation studies.

## Methods

### Experimental design

The experiments described in this paper were conducted in four participating laboratories. Each laboratory used their own cell banks, reagents, cell culture media, and sonication devices.

### Particles

DQ12 quartz silica (Robock 1973) was distributed among participating laboratories by IOM. NM-203 was provided by the European Commission, Joint Research Centre (JRC), JRC Nanomaterials Repository, Ispra, Italy. Each participating laboratory used their own existing stock of NM-203. Silica-Std and Silica-Silane were kindly provided by Nouryon (Sweden, formerly known as AkzoNobel), and each participating laboratory used the same stock. Silica-Silane is Silica-Std with additional 3-(2,3-dihydroxypropoxy)propyl) silanetriol organosilane groups on its surface. 2.35 µmol/m^2^ organosilane was added during the silanization process. Silica-Silane is the same material as used in Ag Seleci et al. (2022) where it is also called Silica-Silane, and in Ruijter et al. (2025) where it is called Silica-Sil3. Particle characteristics are displayed in Table 1. Since DQ12 does not fall within the nano-range, we refer to the materials as particles instead of NMs.

**Table 1:** Particles used in this study and their characteristics

Particle	Type of silica	Production	Primary particle size	Specific surface area (m^2^/g)	Dissolution half time in lung lining fluid (pH 7.4) in days	Dissolution half time in phagolysosome fluid (pH 4.5) in days
DQ12	Quartz (crystalline)	Naturally occurring/fracturing	0.4–1.6 µm (Albrecht et al. 2004)	7.4, BET (Robock 1973)	> 100 estimated	> 100 estimated
NM-203	Pyrogenic amorphous	Flame hydrolysis (thermal)	74 nm (Rasmussen et al. 2013)	204, BET (Rasmussen et al. 2013)	Not assessed	0.4 ng/cm^2^/h (Wohlleben et al. 2019), halftime: 35 days (personal communication with the authors)
Silica-Std	Colloidal amorphous	Wet-chemical process	17 nm (provided by manufacturer)	364, Sears titration (provided by manufacturer)	0.5 (Keller et al. 2021, 2023)	3.9 (Keller et al. 2021, 2023)
Silica-Silane	Silane surface functionalized colloidal	Wet-chemical process	17 nm (provided by manufacturer)	364, Sears titration (provided by manufacturer)	0.8 (Keller et al. 2021, 2023)	3.6 (Keller et al. 2021, 2023)

### Particle characterization

#### Dynamic light scattering

To assess the behaviour of the particles in cell culture medium (CCM), dynamic light scattering (DLS) analysis was performed. The particles were diluted to 100 µg/mL in either RPMI or MEM without phenol red, or 20 mM NaCl or water, of which 1 mL was transferred to a cuvette (Malvern, DTS0012) and placed in the DLS apparatus (Malvern Zetasizer Ultra). Z-average and polydispersity were assessed three times on three fresh dispersions and analysed using ZS Explorer DLS software. Zeta-potential was measured using DTS1070 cuvettes. RPMI 1640 without phenol-red (Gibco, 11835-030) was used as an alternative for RPMI-Glutamax, and MEM without phenol-red (Gibco, 41061-029) was used as an alternative for MEM, with and without 10% foetal bovine serum (FBS) (Gibco, 1050064) supplementation.

#### Optimal sonication procedures

The DeLoid et al. (2017) protocol was followed to determine the optimal sonication procedure to achieve the smallest possible hydrodynamic diameter and most stable dispersion of DQ12 and NM-203. In short, DQ12 and NM-203 powders were weighed and dispersed in ultrapure water to achieve a concentration of 2.5 mg/mL. The dispersions were vortexed for 30 s and the hydrodynamic diameter was assessed using DLS. Subsequently, the dispersions were probe sonicated on ice at 10% amplitude for 1 min and then assessed using DLS again. These steps were repeated until the hydrodynamic diameter did not decrease more than 5%. This process was repeated three times for each particle, and critical delivered sonication energy (crDSE) was calculated from the results, using specific delivered sonication energy (DSE) values that were previously determined for each sonicator following DeLoid et al. (2017). Silica-Std and Silica-Silane were not sonicated as they are stable colloidal dispersions.

#### Deposited dose submerged

The deposited dose of DQ12 was modelled using the DG model in the RiskGone online module (Cheimarios et al. 2022). The DeLoid et al. (2017) protocol and calculations were followed to obtain effective densities of the particles in CCM. In short, the particle dispersions in RPMI 1640 without phenol-red (Gibco, 11835-030) with and without 10% FBS (Gibco, 1050064) supplementation were centrifuged in packed cell volume (PCV) tubes (TPP, Z760986-50EA) at 3000 × g for 1 h and pellet size was determined using the easy-read device (TPP, 87010). Whole size-distributions originating from DLS analysis were fed into the RiskGone module. Deposited doses for NM-203, Silica-Std and Silica-Silane could not be determined by modelling as pellets after centrifugation were either not visible or did not show a clear border. Therefore, the deposited dose of NM-203 was determined following the same procedure as in Ruijter et al. (2025). In short, gelatine gels were made in 96-well plates and dispersions of NMs in exposure medium were added to the wells after gels were set. After 24 h of incubation the two compartments were separated, and Si content was measured in both the gels and the supernatants using inductively coupled plasma optical emission spectroscopy (ICP-OES). The deposited dose of Silica-Std and Silica-Silane were extracted from Ruijter et al. (2025).

#### SEM analysis

The particles that were nebulized in the radial cloud system (explained below) were collected on Carbon Film 300 Mesh TEM grids (Electron Microscopy Sciences, CF300-Cu-25). The highest deposited dose samples were used for analysis. Samples were investigated by scanning electron microscopy (SEM-FEG, LEO 1530, Germany). All the samples were coated with a 10 nm thick carbon layer deposited by sputtering. Images were acquired with a working distance of approximatively 8 mm, an accelerating voltage of 5 kV, a magnification from x15k to x500k and a diaphragm aperture of 30 μm.

### Air–liquid interface experiments

#### NHBE culture conditions

Normal Human Bronchial Epithelial cells (NHBE) from three donors were obtained from Lonza and cultured in PneumaCult Ex Plus Basal medium (Stemcell, Cat #05041) supplemented with PneumaCult Ex plus supplement (Stemcell, Cat #05042), hydrocortisone (Stemcell, Cat #07925), and Penicillin–Streptomycin (Lonza, Cat#17-602E). NHBEs from three donors were individually expanded for one week, dissociated using Trypsin–EDTA (Lonza, cat. CC-5012) for 6 min at 37 °C, counted, and then seeded on inserts (Corning 3460, 12 mm, 0.4 µm pores) coated in rat-tail collagen (ThermoFisher, Cat #A1048301) at a 1:1:1 donor to donor ratio. 2 × 10^5^ cells of the mixture were seeded per insert, using 1.5 mL of Ex Plus medium on the basolateral side and 0.5 mL on the apical side. When cells reached confluency (generally 3–4 days after seeding) the inserts were airlifted (apical medium removed) and the medium was changed to PneumaCult ALI Basal medium (Stemcell, Cat #05001), supplemented with PneumaCult ALI Supplement (Component #05001) and PneumaCult^™^-ALI Maintenance Supplement (Component #05001), hydrocortisone, heparin, and penicillin–streptomycin. Inserts were maintained in basal medium on the basolateral side, medium was refreshed three times per week, and inserts were washed once per week until exposures 32 days later. One day before exposures, trans-epithelial resistance (TEER) was measured using an Evom2 Voltohmmeter equipped with a chopstick electrode (World Precision Instruments), inserts were washed, and basolateral medium was changed to ALI Basal medium without heparin and hydrocortisone.

#### Calu-3 and THP-1 co-culture conditions

The previously optimized PATROLS standard operating procedure (SOP) for culturing and exposures of Calu-3 and THP-1 co-cultures (PATROLS SOP 3406, see also (Braakhuis et al. 2023)) was followed with a few modifications. In brief, Calu-3 cells were maintained in MEM-Glutamax medium (Gibco, 42360–081) supplemented with 10% FBS (Gibco, 1050064), 1% MEM NEAA (Gibco, 11140–035), and 1% Penicillin/Streptomycin (Gibco, 15140122). Human monocyte cell-line THP-1 (TIB-237 202, ATCC) cells were maintained in RPMI-Glutamax (Gibco, 72400–021) supplemented with 10% FBS, and 1% Penicillin/Streptomycin. Calu-3 cells were seeded on inserts (Corning 3460, 12 mm, 0.4 µm pores) on day one at a density of 1 × 10^5^ cells/cm^2^. Inserts were air-lifted on day eight by removing apical medium. On day thirteen, THP-1 cells were differentiated to macrophages on 6-well plates using 10 ng/mL Phorbol 12-myristate 13-acetate (PMA) (Invivogen tlrl-pma), at a density of 5 × 10^5^ cells per well (5.21 × 10^4^ cells/cm^2^) for 24 h. After 24 h, cells were washed twice with HBSS and fresh CCM was added. After 24 h of recovery (on day fifteen), differentiated THP-1 (dTHP-1) cells were detached by incubating them for 50 min in 1 mL cold accutase (Gibco A11105) per well at room temperature. dTHP-1 cells were added to each freshly washed insert at a density of 5 × 10^4^ cells/cm^2^ in 0.5 mL CCM and incubated at 37 °C and 5% CO_2_ to attach for 4 h, after which the apical medium was carefully removed. On day sixteen cells were exposed (giving the dTHP-1 cells an effective recovery time of 48 h) and on day seventeen the responses were analysed (see below).

#### Deviations from PATROLS SOP 3406

The differentiation protocol and seeding densities of dTHP-1 cells on the inserts were different from PATROLS SOP 3406. Several differentiation protocols (PMA concentrations, differentiation durations and recovery durations) were evaluated and the protocol that resulted in the highest cytokine response (TNF-α) to lipopolysaccharide (LPS), while still maintaining low baseline cytokine levels was chosen. The seeding density of dTHP-1 cells on the Calu-3 cells was four times higher than in PATROLS SOP 3406 as fluorescent staining (by Vybrant^™^ DiO Cell-Labelling Solution, Invitrogen, V22886, following manufacturer’s instructions) of dTHP-1 cells showed substantial loss of cells after the 4 h attachment step. The adapted seeding density resulted in a good amount of dTHP-1 cells on the inserts upon fluorescent microscopy as shown in Supplementary Figure S1. Propidium Iodine stain (12.5 µg/mL in CCM, 200 µL per insert apically) was used to visualize cell death.

#### Exposures at the ALI

The PATROLS SOP for NM aerosolization (PATROLS SOP 3601) was followed for cloud exposures of inserts with NHBE or Calu-3-THP-1 co-cultures, with a few modifications. DQ12 and NM-203 were weighed, diluted to 2.5 mg/mL in ultrapure water (B. Braun, 3624390), and sonicated using probe sonication for 1:40 and 2:20 min respectively at 10% amplitude. The silica particle dispersions were diluted in ultrapure water (B. Braun, 3624390), containing 0.009% NaCl to facilitate nebulization of the dispersions. A radial in vitro aerosol exposure system (RIVAES, designed by RIVM, inspired by the design of the VITROCELL Cloud exposure system (Vitrocell, Waldkirch, Germany)) was used for the exposures. In the RIVAES, the transwell inserts are placed radially to minimize variation in deposition. The system has a slightly smaller redundant surface area than the VITROCELL^®^ Cloud system, resulting in a slightly higher deposition on the cells. It is equipped with a refined temperature control system resulting in a stable temperature at the transwell inserts. A 4–6 µm pore size vibrating mesh nebulizer was used (Vitrocell). DQ12 and NM-203 particles were diluted to 500 μg/mL and nebulized 2x, 4x, or 6x to reach required deposited doses. Higher concentrations of these larger particles could block the nebulizer. Silica-Std and Silica-Silane were diluted to 1, 2, and 3 mg/mL to reach required deposited doses and nebulized once. One nebulization of 200 µL of 175 µg/mL LPS (Sigma Aldrich L4391) was used as a positive control. A Quartz Crystal Microbalance (QCM) (Vitrocell) was used to determine depositions of exposures following PATROLS SOP 3601, but with optimized timing allowing the signal to fully stabilize before and after nebulizations.

#### Harvest and analyses

24 h after exposures, 500 µL culture medium without FBS was added apically and left for exactly 10 min, after which barrier integrity was confirmed using TEER. Apical and basolateral supernatants were collected in Eppendorf tubes, kept on ice, and centrifuged for 15 min at 10.000 × g at 4 °C to remove NMs and cell debris. 100 µL of this supernatant was directly used for lactate dehydrogenase (LDH) analysis following manufacturer’s instructions (Roche, 11644793001), and the remaining volume was stored at −80 °C for cytokine analysis. Cytokine release was measured using ELISA according to manufacturer’s instructions for IL-6 (ThermoFisher, 88-7066-88), IL-8 (88-8086-88), MCP-1 (88-7399-88), and TNF-α (88-7346-88).

### Submerged system experiments—epithelial cells

#### Cytokine release and viability A549 and Calu-3 cells

Calu-3 and A549 cells were cultured in MEM (Sigma Aldrich M0446) supplemented with 10% FBS (Gibco 10270-106) and 1% Penicillin/Streptomycin (Gibco 15140-122). Calu-3 and A549 cells were seeded in 96-well plates at 1.25 × 10^5^ cells/cm^2^ in CCM on day one. On day four, DQ12 and NM-203 were weighed, diluted to 1.5 mg/mL in ultrapure water and sonicated using probe sonication for 1:40 and 2:20 min, respectively. Next, the silica particles were diluted in CCM without FBS, and 0.2 mL was applied to the cells for 24 h. LPS at 100 ng/mL was used as a positive control. On day five, CCM was collected and frozen at −20 °C until analysis. Cells were incubated with 0.1 mL of MTT working solution per well for 1–2 h, until cells were all stained. After the incubation time, the remaining medium was carefully removed and 0.1 mL of dimethyl sulfoxide (DMSO 99.5%, PanReac AppliChem 161954.1612) was added to each well. Plates were agitated for 10 min until DMSO homogenized the cells. Absorbance was read at 570 nm. Secretion of IL-6, IL-8, and TNF-α was analyzed using the DuoSet ELISA Ancillary Reagent Kit DY007B (R&D Systems) + Human IL-6/IL-8 DuoSet ELISA (DY-206-05/DY208-05 R&D Systems).

#### Gene expression and viability of BEAS-2B and A549 cells

BEAS-2B and A549 cells were cultured in DMEM Medium with Glutamax (Gibco 319660-21) supplemented with 10% FBS and 1% penicillin/streptomycin. BEAS-2B cells were grown on collagen-coated plates (0.03 mg/mL bovine collagen type I) and seeded at 37,500 cells/cm^2^ in collagen-coated 24-well plates. A549 cells were seeded at 75,000 cells/cm^2^ in uncoated 24-well plates. The next day, silica particles were weighed, diluted to 2.56 mg/mL in ultrapure water and sonicated using probe sonication for 5 min at 10.2 W. Particles were diluted in CCM without FBS and 625 µL was applied to the cells at one dose that was established to be subtoxic: 32 µg/mL for A549 cells and 16 µg/mL for BEAS-2B cells. After 24 h of exposure, cells were washed three times with PBS and lysed using the mRNA assay kit lysis reagent (GeneElute total RNA miniprep kit, Merck Sigma-Aldrich, RTN70) using the supplier’s procedure. Lysed cells were stored at −80 °C until extraction. Total RNA was extracted, quality checked and reverse transcribed using superscript III reverse transcriptase (ThermoFisher Scientific, 18080044). The obtained cDNA was used for qPCR, which was performed using Takyon No ROX SYBR Master Mix blue dTTP (Eurogentec, UF-NSMT-B0710) on a CFX96 Touch system (Biorad) and the following thermal profile: 5 min at 95 °C, then 40 cycles of [15 s at 95 °C, 20 s at 55 °C, 40 s at 72 °C], then 1 min at 95 °C, 30 s at 55 °C and 30 s at 95 °C for obtaining the dissociation curve. The used primer sequences were IL-1β (F 5′-ACAGATGAAGTGCTCCTTCCA-3′, R 5′-GTCGAGATTCGTAGCTGGAT-3′); MCP-1 (F 5′-CATTGTGGCCAAGGAGATCTG-3′, R 5′-TTCGTTTCCCTTTGAGGCTTC-3′); IL-8 (F 5′-GAATGGGTTTGCTAGAATGTGATA-3′, R 5′-CAGACTAGGGTTGCCAGATTTAAC-3′); TNF-α (F 5′-GAGCAGTGAAAGCATGATCC-3′, R 5′-CGAGAAGATGATCTGACTGCC-3′); IL-6 (F 5′-CCTCGACGGCATCTCAGCCCT, R 5′-TCTGCCAGTGCCTCTTTGCTGC-3′). For viability analysis, the same seeding densities were used, but in 96-well plates. The same particle preparation and exposure procedure was followed, but with 100 µL per well of serially diluted exposures. Viability was assessed after 24 h using WST-1 (Merck Sigma Aldrich, 5015944001) following manufacturer’s instructions.

### Submerged system experiments—macrophages

#### Cytokine release—M0 vs M1 macrophages

Human monocyte THP-1 cells were maintained in RPMI 1640 medium with 10% FBS, 100 U/ml penicillin and 100 μg/mL streptomycin (Thermo Fisher Scientific, UK) and were differentiated according to the protocol of Genin et al. (2015). For M0 macrophages, cells were seeded at 1.8 × 10^4^ cells/cm^2^ in 96-well plates in CCM containing 92.5 ng/mL PMA and incubated for 24 h, after which cells were washed and rested in fresh CCM 24 h before treatment. For M1 cells, after the 24 h rest period used for M0 cells, IFN-γ at 20 ng/mL and LPS at 10 pg/mL were added for 24 h, cells were then washed, and particle treatments were applied. Prior to each 24 h treatment, DQ12 and NM-203 were suspended at 1 mg/mL, vortexed and sonicated in an Ultrawave QS25 sonicating water bath for 10 min (operating at 400 J/s) prior to serial dilutions. Silica-Std was diluted to 1 mg/mL in CCM and then serial diluted. The exposure volume was 0.1 mL in CCM containing FBS. Triton X-100 at 0.1% v/v and LPS at 100 and 1000 ng/mL were used as positive controls for cytotoxicity and pro-inflammatory response, respectively. Alamar Blue reagent (Thermo Fisher Scientific, UK) was used to assess cell viability. A 1.25% (v/v) solution was prepared in FBS-free, phenol red-free MEM 24 h before use. After treatment, cells were washed, 100 µL Alamar Blue reagent was added, and incubated for 120 min protected from light. Fluorescence intensity was read at excitation/emission wavelengths of 532/590 nm for resorufin using a Tecan Spark 10 M plate reader. Secretion of pro-inflammatory cytokines IL-1β and IL-6 was assessed using ELISA (Invitrogen™, ThermoFisher), following the manufacturer’s guidelines, with absorbance read using a Tecan Spark 10 M plate reader.

#### IL-1β release from dTHP-1 following REFINE SOP

Secretion of IL-1β and cell viability were assessed using the previously optimized REFINE SOP (Vandebriel et al. 2022). THP-1 cells (TIB-237 202, ATCC) were grown in RPMI-Glutamax (Gibco, 72400-021) supplemented with 10% FBS (Gibco, 1050064) and 1% Penicillin/Streptomycin (Gibco, 15140122). CCM was refreshed twice per week by centrifugation at 130 × g for 5 min, discarding the culture medium, counting the cells, and adding fresh culture medium at a concentration of 2 × 10^5^ cells/mL. THP-1 cells were differentiated into M0 macrophages (dTHP-1) by adding 100 ng/mL PMA (InvivoGen, tlrl-pma) to 5 × 10^5^ cells/mL and plating 100 µL of cell suspension to each well of a 96-well plate (1.56 × 10^5^ cells/cm^2^). The THP-1 cells were incubated with PMA for 3 h which allowed the cells to attach to the wells. After 3 h, medium was removed, fresh CCM was added, and the cells were incubated at 37 °C for 20–24 h. DQ12 and NM-203 were weighed, diluted to 2.5 mg/mL in ultrapure water (B. Braun, 3624390), and sonicated using probe sonication for 1:40 and 2:20 min respectively at 10% amplitude. Next, the silica particles were diluted in CCM containing 10% FBS and added to the wells (200 µL per well) in concentrations ranging from 1.56 to 100 µg/mL. Nigericin (0.625 and 1.25 mM) was used as a positive control. The cells were incubated for 48 h at 37 °C and 5% CO_2_. After incubation, cell viability was assessed using WST-1 proliferation reagent (Roche, 5015944001) according to manufacturer’s instructions and IL-1β production was measured using ELISA according to manufacturer’s instructions (Invitrogen, 88-7261-88). Activation of the NLRP3 inflammasome was not confirmed with NLRP3 deficient THP-1 cells.

#### dTHP-1 submerged following the same differentiation procedure as ALI experiments

To compare ALI responses to submerged responses, THP-1 cells were differentiated following the same protocol as was used for ALI exposures but exposed in submerged monocultures. THP-1 cells were cultured in RPMI 1640 (Sigma Aldrich M4130) supplemented with 10% FBS (Gibco 10270-106), 0.05 mM β-mercaptoethanol (2-mercaptoehtanol 98% Sigma M3148), and 1% Penicillin/Streptomycin (Gibco 15140-122). Cells were seeded at 5 × 10^4^ cells/cm^2^ (same seeding density as in co-cultures at ALI) in CCM containing 10 ng/mL PMA (Sigma P1585) in 96-well plates. THP-1 cells were kept in PMA-containing CCM for 24 h, after which they were put on fresh CCM for another 48 h. On day four, DQ12 and NM-203 were weighed, diluted to 1.5 mg/mL in ultrapure water and sonicated using probe sonication for 1:40 and 2:20 min, respectively. Next, the silica particles were diluted in CCM without FBS, and 0.2 mL was applied to the cells and incubated for 24 h. LPS at 100 ng/mL was used as a positive control. On day five, CCM was collected and frozen at −20 °C until analysis. Cells were incubated with 0.1 mL of MTT working solution per well for 1–2 h, until cells were all stained. After the incubation time, the remaining medium was carefully removed and 0.1 mL of dimethyl sulfoxide (DMSO 99.5%, PanReac AppliChem 161954.1612) was added to each well. Plates were agitated for 10 min until DMSO homogenized the cells. Absorbance was read at 570 nm. IL-6, IL-8, and TNF-α were measured using the DuoSet ELISA Ancillary Reagent Kit DY007B (R&D Systems) + Human IL-6/IL-8 DuoSet ELISA (DY-206-05/DY208-05 R&D Systems).

#### DCFH assay

The potential of the silica particles to induce ROS intracellularly was assessed using the previously optimized flow cytometry-based DCFH assay (Ruijter et al. 2024b). RAW276.4 cells were maintained in DMEM/F-12 GlutaMax (Gibco, 31331-028), supplemented with 10% FBS (Gibco, 1050064), 1% sodium pyruvate MEM 100 mM (Gibco, 11360-070), and 1% Penicillin/Streptomycin (Gibco, 15140122). On day one, cells were seeded on 24-well plates at a density of 1 × 10^5^ cells/cm^2^ and were allowed to attach overnight. On day two, cells were incubated with 6 µg/mL CM-H_2_DCFDA (Invitrogen, C6827) for 30 min in the dark at 37 °C and 5% CO_2_, after which the cells were washed and incubated with 500 µL particle treatments diluted in CCM without FBS. DQ12 and NM-203 were weighed, diluted to 2.5 mg/mL in ultrapure water (B. Braun, 3624390), and sonicated using probe sonication for 1:40 and 2:20 min respectively at 10% amplitude. After 24 h of incubation, cells were detached by scraping, centrifuged, incubated with Fixable viability stain 780 (BD Horizon, 565388) according to manufacturer’s instructions, washed by centrifugation and resuspended in cold HBSS. Flow cytometry analysis was conducted immediately afterwards using the BD Biosciences Fortessa Cell Analyzer. Singlets and alive cells were gated for using the APC-Cy7 channel and median fluorescence intensity (MFI) for DCF was recorded using the FITC channel. 10,000 events were collected within the alive gate. Settings were kept constant for all analyses. Aminated polystyrene nanoparticles of 60 nm were used as positive control (Magsphere, AM060 NM). Results were expressed as fold change in fluorescence as compared to the negative control.

### Reactive oxygen species

#### FRAS assay

The oxidative potential of the silica particles was assessed using the ferric reducing ability of serum (FRAS) assay, which was previously optimized (Ruijter et al. 2024a). In short, particles were weighed in glass vials and incubated with human blood serum (HBS, Sigma P2918-100 ml) for 3 h at 37 °C shaking at 150 rpm after mixing thoroughly using 1 min bath sonication. Samples were then centrifuged at 14,000 × g for 150 min, and supernatants were transferred to 96-well plates. FRAS reagent was added to the 96-well plates and absorbance was measured at 593 nm after 1 h of incubation. CuO NMs (PlasmaChem, YF131107) were used as positive control, and NM-220 BaSO_4_ NMs (JRC, Ispra) were used as negative control particles.

#### Haemolysis assay

The red blood cell (RBC) haemolysis protocol developed and used in Ruijter et al. (2025) was followed. Briefly, particles were diluted in PBS to 15 mg/mL, dispersed using vortexing and bath sonication for 10 min, and concentration ranges were made in PBS. 0.1% Triton-X was used as 100% haemolysis condition. Whole sheep blood in Alsever’s solution (Thermo Scientific, 12977755) was washed twice by adding 1 mL PBS to 1 mL blood and centrifuging for 5 min at 250 × g. After the second wash, pellets were resuspended in 3.6 mL cold PBS each, combined and kept on ice until use. Particle suspensions and red blood cells were combined in flat-bottomed 96-well plates and agitated at room temperature for 10 min. Plates were then centrifuged for 15 min at 250 × g, supernatant was transferred to a clean 96-well plate and absorbance was measured at wavelength 540 nm, with interference 650 nm. Results were expressed as % haemolysis compared to the 0.1% Triton-X condition.

### Data analysis and in vitro ranking

Each assay was conducted a minimum of three independent times, containing at least three technical replicates except for the following experiments: The DCFH assay was carried out with two technical replicates per experiment; Gene expression experiments were conducted once. qPCR data were analysed using the Relative Expression Software Tool (Data were analysed using the ΔΔCq method using the Relative Expression Software Tool (REST) (Pfaffl 2001). For the other assays, averages of each independent experiment were analysed by one-way ANOVA, followed by Dunnett’s multiple comparisons test, using GraphPad Prism version 9.5.1. Rankings were established using benchmark dose (BMD) modelling using the PROAST web version 70.1. Data were modelled using Exponential and Hill models and the lowest BMD confidence interval lower limit (BMDL) and highest BMD confidence interval upper limit (BMDU) are reported. Different critical effect sizes (CES) were used per assay and were derived by determining the fold change required to reach half the maximum response observed in that assay.

## Results

### NM characterization and deposited dose

DQ12, NM-203, Silica-Std, and Silica-Silane were included as case study materials to represent crystalline (quartz), pyrogenic SAS, colloidal, and silane functionalized colloidal silica particles, respectively. The characteristics of the four silica case study materials in three cell culture media and in saline water, and the optimal sonication energy following DeLoid et al. (2017) are shown in Supplementary Table S1. Scanning electron microscopy (SEM) images are shown in Supplementary Figure S1. Table 2 shows deposition efficiencies of the silica particles in cell culture medium (CCM) with and without 10% foetal bovine serum (FBS) supplementation in a 24 h submerged experiment. NM-203, Silica-Std, and Silica-Silane depositions could not be modelled in silico as no clear pellet was formed during the crucial centrifugation step for determining effective density and were therefore assessed using inductively coupled plasma optical emission spectroscopy (ICP-OES) following previously established methods (Ruijter et al. 2025). The deposited doses of DQ12, NM-203 and Silica-Std in FBS-free conditions were considered identical enough to use the average deposited dose for comparative assessment: 54.9% ± 3.4. The deposited doses in FBS-containing conditions were not considered equal and the deposition efficiencies in Table 2 were used to calculate deposited doses for each particle separately, extrapolating the deposition efficiency to all administered doses. FBS supplementation increased deposition efficiency of Silica-Std from 58 ± 8.6% without to 86.6 ± 1% with FBS supplementation but did not affect the deposition efficiency of NM-203 (Table 2). Z-average of Silica-Std increased from 22 ± 1.9 nm without FBS to 74.7 ± 2.1 nm with FBS supplementation (Supplementary Table S1). Z-averages of NM-203 an DQ12 decreased slightly upon FBS supplementation (573.3 ± 11.3 nm to 401.2 ± 23.8 nm for DQ12 and 167.8 ± 2.6 nm to 137.3 ± 3.7 nm for NM-203) (Supplementary Table S1). The deposited dose of Silica-Silane was considered zero and this particle could therefore only be assessed using non-adherent cell types under agitation or at the ALI where cells are exposed to particle-containing liquid aerosols on the apical side.

**Table 2:** Deposition efficiency of silica particles in 96-well plates at 100 µg/mL after 24 h

Particle	Deposition efficiency without FBS (%) ± SD	Deposition efficiency with FBS (%) ± SD	Method
DQ12	51.5	41.1	DG model (RiskGone module)
NM-203	55.3 ± 8.8	55.2 ± 8.9	ICP-OES
Silica-Std	58.0 ± 8.6	86.3 ± 1.0	ICP-OES, results extracted from Ruijter et al. (2025)
Silica-Silane	10.2 ± 15.9 (considered zero)	−5.2 ± 31.6 (considered zero)	ICP-OES, results extracted from Ruijter et al. (2025)

*FBS* foetal bovine serum, *DG* distorted grid, *ICP-OES* inductively coupled plasma optical emission spectroscopy

### Air–liquid interface

**Fig. 1:**
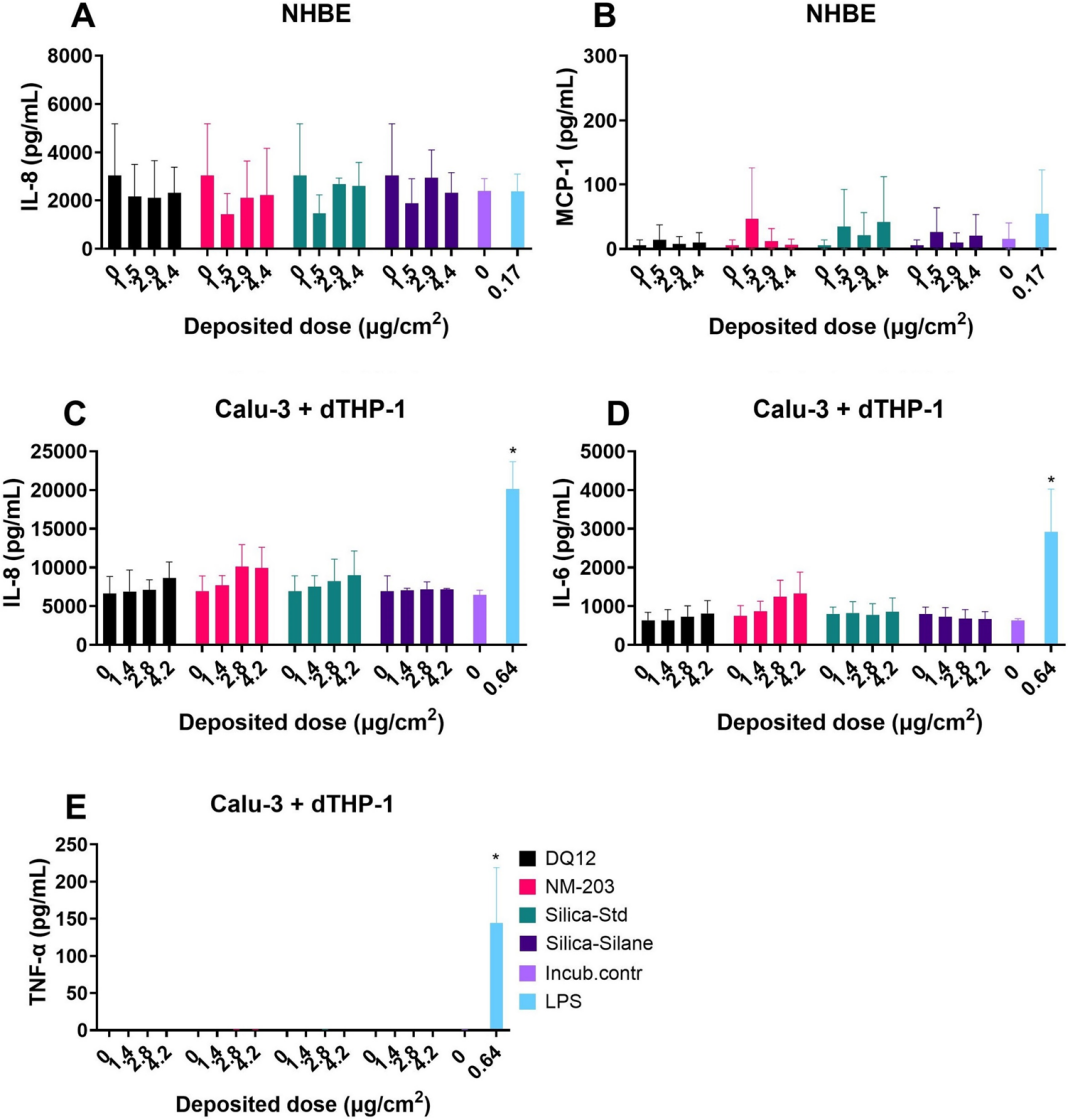
Cytokine secretion at the air–liquid interface after 24 h particle exposures using RIVAES, as measured using ELISA in the apical compartment. Normal Human Bronchial Epithelial cells obtained from donor material (**A** + **B**) and Calu-3 + dTHP-1 co-cultures, following PATROLS SOP with minor modifications (**C** + **D** + **E**). Results are the average of three independent experiments. *p < 0.05 as analysed by one-way ANOVA and post-hoc analysis as compared to the corresponding 0 µg/cm^2^ control condition

Secretion of cytokines relevant for pulmonary inflammation by normal human bronchial epithelial (NHBE) cells without added macrophages, and by Calu-3 bronchial epithelial cells in co-culture with differentiated THP-1 (dTHP-1) macrophages in response to 24 h exposures to the four SiO_2_ particles at the ALI using aerosolization in a radial in vitro aerosol exposure system (RIVAES) is shown in Fig. 1. The SiO_2_ particle exposures of up to 4.4 µg/cm^2^ did not induce any pro-inflammatory cytokine release in either cell model in the basolateral or the apical compartments (basolateral compartment results not shown). Cytokine secretion by NHBE cells showed relatively large variation between experiments compared to those observed for the co-culture model, even though the same pooled donor material was used for each experiment. A response to LPS was only observed for Calu-3 with dTHP-1 cells and not for NHBE cells. dTHP-1 cells remained viable at the ALI for at least 48 h after the creation of the co-culture (See Supplementary Figure S2). No cytotoxicity (LDH release) or reduction in TEER were observed for either cell model in response to the SiO_2_ particles at the doses tested. The response to the vehicle control (0 µg/cm^2^ condition) did not differ from the response in the incubator control condition, meaning that the cells did not show pro-inflammatory responses towards being transferred to and exposed inside the RIVAES.

### Submerged cell models – epithelial cells

**Fig. 2:**
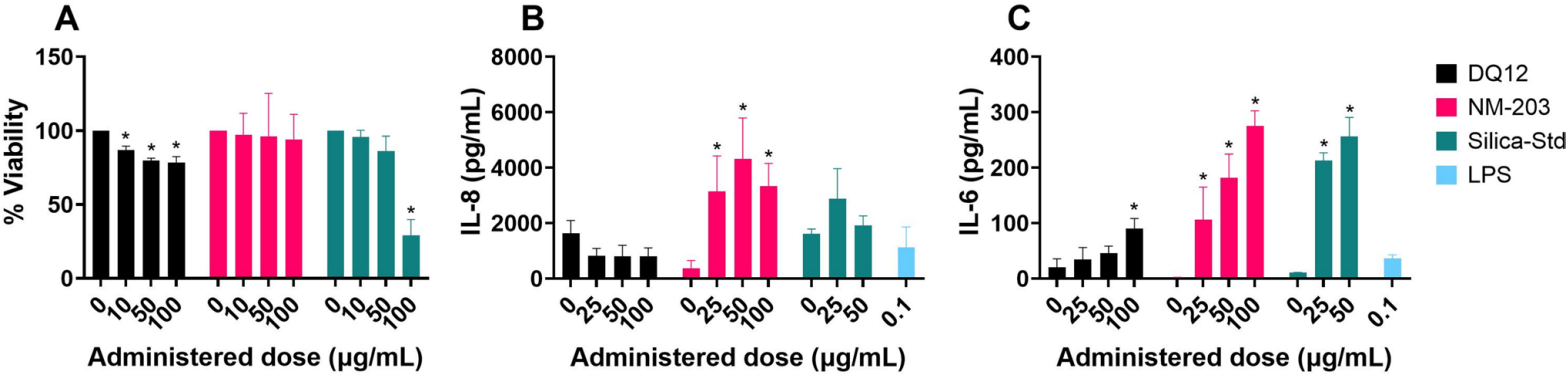
Cell viability by means of MTT assay (**A**), IL-8 secretion (**B**), and IL-6 secretion (**C**) by means of ELISA in Calu-3 cells after submerged exposure to SiO_2_ particles for 24 h. Cytokine secretion at doses with reduced cell viability are not shown. Results are the average of three independent experiments. *p < 0.05 as analysed by one-way ANOVA and post-hoc analysis as compared to the corresponding 0 µg/mL control condition

Calu-3 bronchial epithelial cells were exposed to dose-ranges of DQ12, NM-203 and Silica Std and cell viability and secretion of pro-inflammatory cytokines IL-8 and IL-6 were assessed (Fig. 2). The response of Silica-Silane could not be assessed in a submerged setup as it did not reach cells at the bottom of the well (Table 2). Release of IL-6 was induced by DQ12, NM-203, and Silica-Std. The release of IL-8 was less consistent across silica particles tested, with only NM-203 inducing significant responses. Dose–response curves are shown in Supplementary Figure S3. Dose–response curves of cell viability of A549 alveolar epithelial cells and Calu-3 cells assessed by MTT assay are shown in Supplementary Figure S4. Cytokine secretion at cytotoxic doses could be related to non-specific effects and are therefore not included in Fig. 2.

**Fig. 3:**
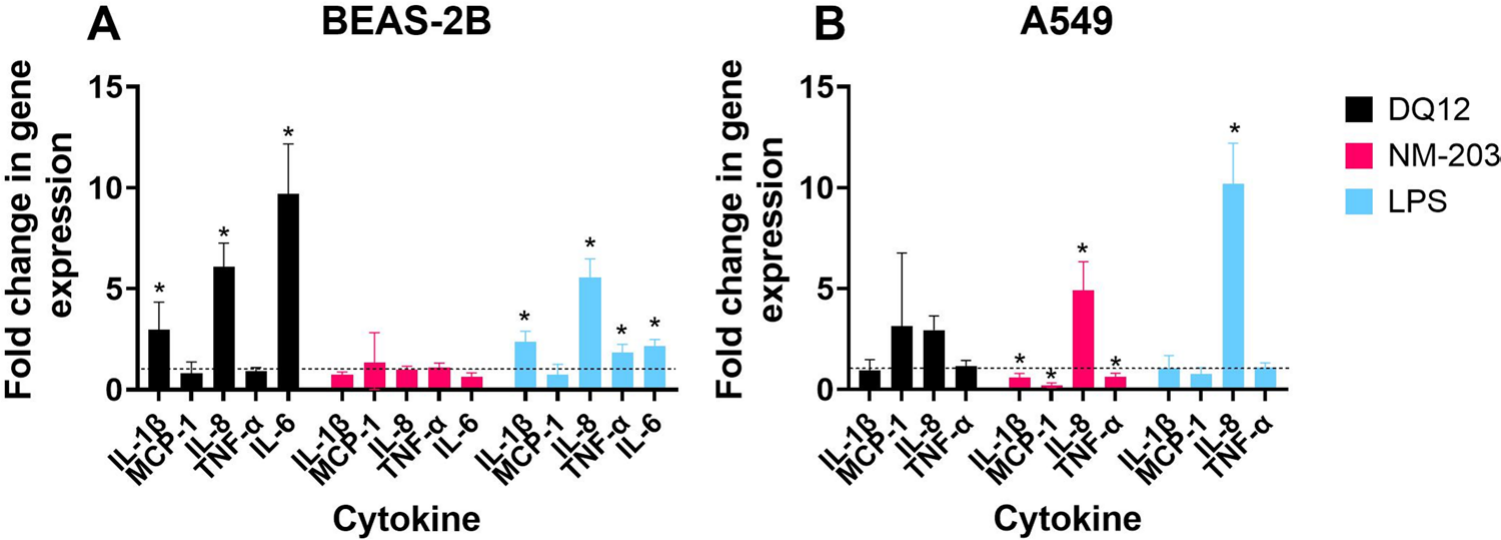
Analysis of pro-inflammatory cytokine gene expression by qPCR in BEAS-2B cells (**A**) and A549 cells (**B**) in a submerged exposure setting. Exposure doses were 16 µg/mL (2.7 µg/cm^2^) for BEAS-2B cells and 32 µg/mL (5.5 µg/cm^2^) for A549 cells and were confirmed to be subtoxic. LPS (250 ng/mL) was used as a positive control. Dotted line represents onefold change in gene expression, thus no change. Experiment was carried out once. *p < 0.05 in REST statistical analysis, means either significantly up- or downregulated as compared to negative control

BEAS-2B bronchial epithelial cells and A549 cells were exposed to one subtoxic dose of DQ12 and NM-203. Gene expression of pro-inflammatory cytokines was assessed after 24 h of exposure (Fig. 3). DQ12 induced pronounced expression of genes encoding IL-1β, IL-8 and IL-6 in BEAS-2B cells, but not in A549 cells. NM-203 induced no response in BEAS-2B cells, while significant upregulation of IL-8 gene expression in A549 cells was observed. Dose–response curves of cell viability of BEAS-2B and A549 cells assessed by WST-1 assay are shown in Supplementary Figure S5.

### Submerged cell models – macrophages

**Fig. 4:**
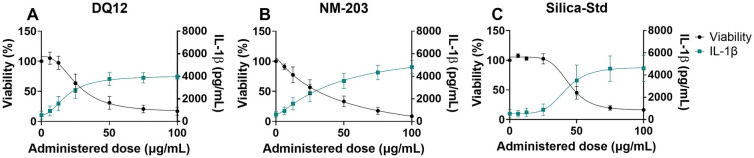
Cell viability and IL-1β secretion in the NLRP3 inflammasome activation assay after 48 h particle exposure. Cell viability was assessed using WST-1 assay (plotted to the left y-axis) and IL-1β secretion (plotted to the right y-axis) was assessed using ELISA. Results are the average of three independent experiments. Curves were fitted using a four-parameter variable slope

The Vandebriel et al. (2022) NLRP3 inflammasome activation assay using dTHP-1 cells as a model for M0 macrophages showed IL-1β release and a corresponding reduction in cell viability in response to the SiO_2_ particles tested, which could point to NLRP3 inflammasome activation (Fig. 4). However, the particles have not been tested using NLRP3 deficient THP-1 cells, as was recommended in the SOP (Vandebriel et al. 2022), and therefore inflammasome activation remains to be confirmed. The dose–response curves of DQ12 and NM-203 are shifted slightly to the left as compared to Silica-Std based on administered doses. PROAST dose–response curves for IL-1β both in correlation to administered as well as to deposited doses are shown in Supplementary Figure S6. Cell viability dose–response curves as measured using WST-1 with and without FBS are shown in Supplementary Figure S7.

**Fig. 5:**
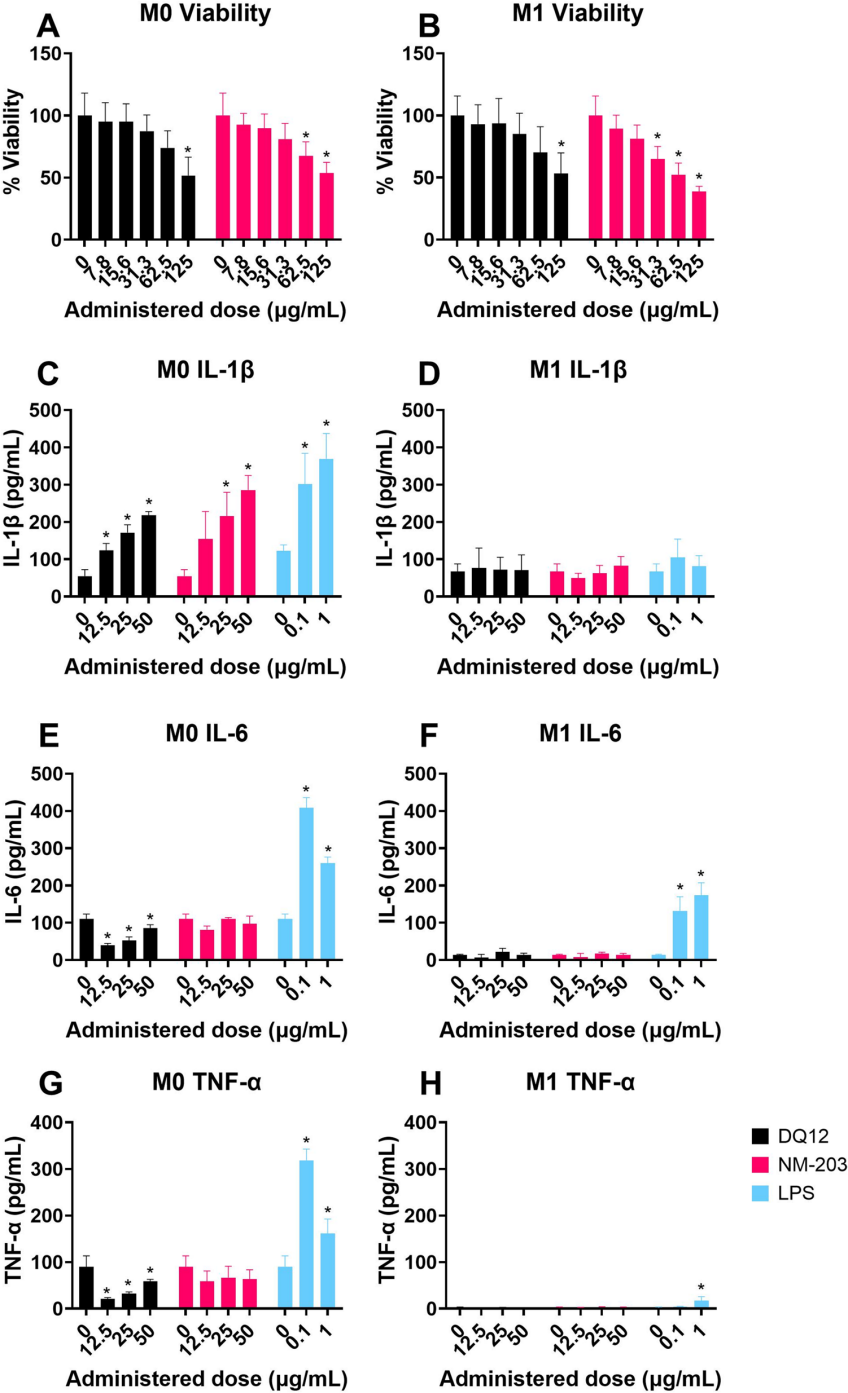
Cell viability (**A** + **B**), IL-1β (**C** + **D**), IL-6 (**E** + **F**), and TNF-α (**G** + **H**) secretion in M0 (**A** + **C** + **E** + **G**) and M1 (**B** + **D** + **F** + **H**) dTHP-1 cells in response to DQ12 and NM-203 after 24 h submerged exposure, as assessed using Alamar Blue and ELISA. Results are averages of three independent experiments. *p < 0.05 as analysed by one-way ANOVA and post-hoc analysis as compared to the corresponding 0 µg/mL control condition

To assess the effect of SiO_2_ particles on M0 versus M1 macrophages and the effect of macrophage subtype on silica particle toxicity ranking, THP-1 cells were differentiated to M1 macrophages using PMA and subsequent IFN-γ and LPS treatment, following Genin et al. (2015). M0 macrophages obtained using the same PMA differentiation protocol, but without IFN-γ and LPS treatment, served as the control group. IL-1β was released by M0 dTHP-1 cells, but not by M1 dTHP-1 cells in response to 24 h exposure to DQ12 and NM-203 (Fig. 5). IL-6 and TNF-α showed no increase in release in either macrophage subtype and are therefore likely not good markers for SiO_2_ toxicity in theses cell models.

**Fig. 6:**
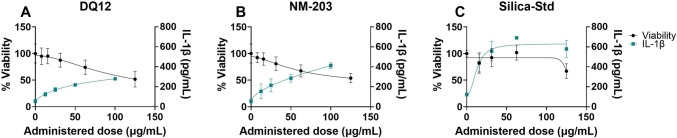
Differentiated THP-1 M0 (Following Genin et al. (2015)) cell viability assessed using Alamar Blue (plotted to the left y-axis) and IL-1β secretion (plotted to the right y-axis) assessed using ELISA in response to 24 h particle exposures. Results are the average of three independent experiments (except IL-1β data of Silica-Std at concentrations of 62.5 and 125 µg/mL; instrumentation error resulted in only two replicates being available for these data points)

For the purpose of establishing hazard rankings, IL-1β secretion and cell viability were assessed for the three SiO_2_ particles which could be tested in a submerged setting in M0 dTHP-1 cells following the Genin et al. (2015) protocol (Fig. 6). Comparing Fig. 4 and Fig. 6 it becomes evident that exposure duration (48 h in Fig. 4 vs 24 h in Fig. 6) and M0 differentiation protocol (Vandebriel et al. (2022) in Fig. 4 vs Genin et al. (2015) in Fig. 6) have an effect on points of departure for both IL-1β secretion as well as cell viability. PROAST dose–response data of IL-1β secretion and cell viability can be found in Supplementary Figures S8 and S9, respectively. PROAST dose–response data of viability of dTHP-1 cells following the same differentiation procedure as was used for ALI co-cultures is shown in Supplementary Figure S10.

**Fig. 7:**
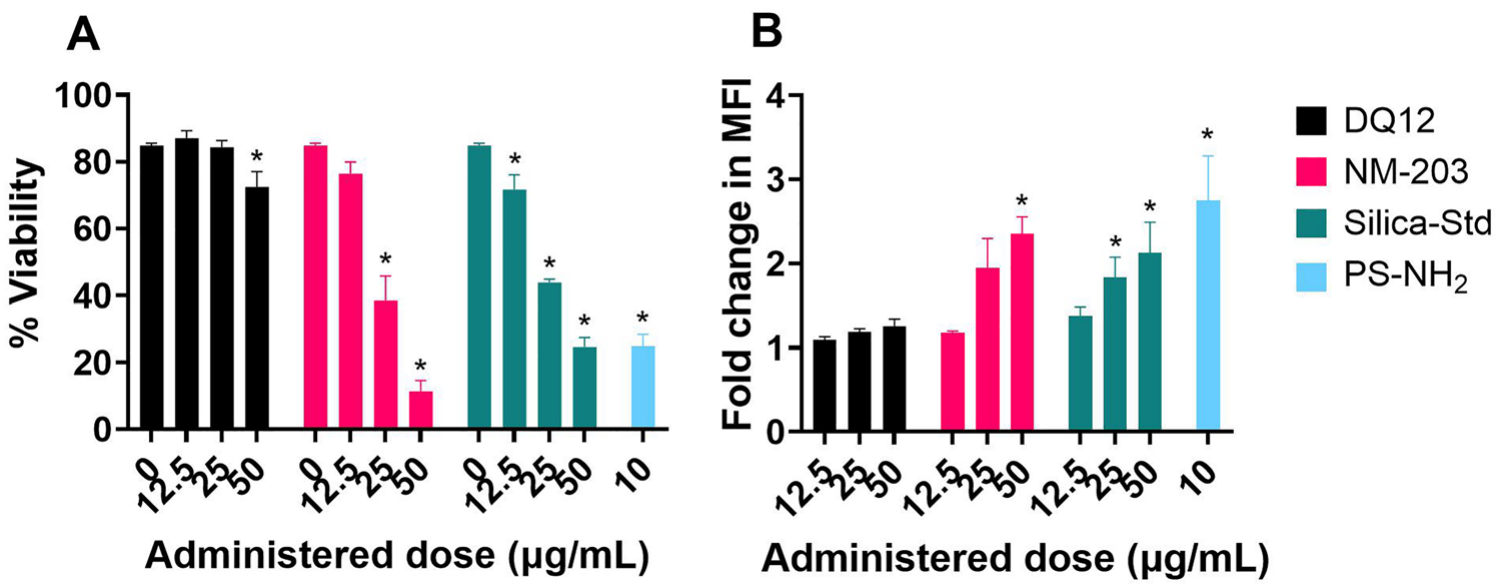
Intracellular ROS in RAW246.7 murine macrophages as assessed using the flow cytometry-based DCFH assay. Results show cell viability (**A**), and fold change in MFI inside alive gate (**B**). Aminated polystyrene NMs (PS-NH_2_) were used as positive control. Results are averages of three independent experiments. *p < 0.05 as analysed by one-way ANOVA and post-hoc analysis on raw fluorescence values as compared to the corresponding 0 µg/mL control condition

Intracellular ROS generation was assessed using RAW246.7 murine macrophages (Fig. 7) using the previously optimized flow cytometry based DCFH assay (Ruijter et al. 2024b). Both NM-203 and Silica-Std induced significant levels of cell death in RAW246.7 cells, with concomitant increase in ROS. DQ12 induced neither. The reported ROS production was assessed in the alive-gate only and is therefore not affected by the reduced viability which is shown in Fig. 7A. Dose–response curves for median fluorescence intensity (MFI) and cell viability can be found in Supplementary Figure S11 and S12, respectively.

### RBC haemolysis assay

**Fig. 8:**
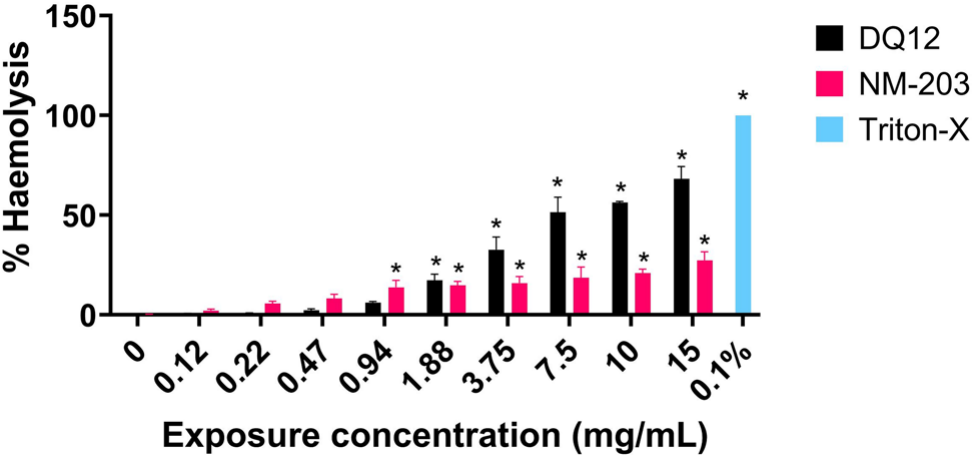
Haemolysis expressed as % haemolysis relative to the 0.1% Triton-X positive control. Results are the average of three independent experiments. *p < 0.05 as analysed by one-way ANOVA and post-hoc analysis as compared to corresponding 0 mg/mL exposure concentration condition

RBC haemolysis was induced by DQ12 and NM-203 (Fig. 8) and not by Silica-Std and Silica-Silane ((Ruijter et al. 2025); Supplementary Figure S13). The NM-203 haemolytic effect had an onset at a lower concentration, but the DQ12 response was more pronounced.

### Acellular FRAS Assay

**Fig. 9:**
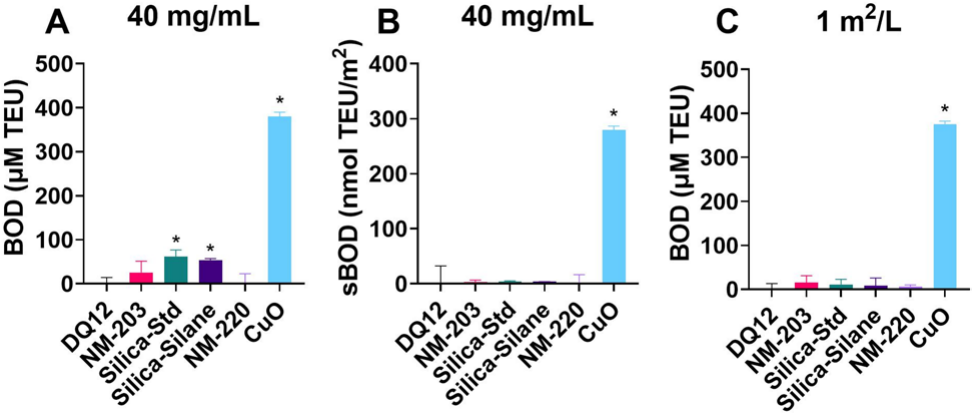
Depletion of human serum antioxidants in response to silica particles as assessed using the FRAS assay following previously optimized procedures. NM-220 BaSO_4_ was used as negative control. CuO NM was used as positive control. **A** 40 mg/mL dose results expressed as biological oxidative damage (BOD), **B** 40 mg/mL dose results expressed as surface area corrected BOD (sBOD), **C** 1 m^2^/L exposure dose expressed as BOD. Results are the average of three independent experiments. *p < 0.05 as analysed by one-way ANOVA and post-hoc analysis as compared to negative control condition (NM-220)

The FRAS assay results, assessed following the SOP published in Ruijter et al. (2024a), show depletion of human blood serum antioxidants by Silica-Std and Silica-Silane, and not by DQ12 and NM-203 (Fig. 9). At the same mass-based dose (40 mg/mL) there was a small but significant effect of Silica-Std and Silica-Silane (Fig. 9A). However, when corrected for surface area (Fig. 9B), and when tested the same surface-based dose (1 m^2^/L, Fig. 9C) the effect is no longer significant, indicating that the effect of Silica-Std and Silica-Silane seems to be due to their large surface area (364 m^2^/g).

### Hazard ranking based on data derived from in vivo studies

There is strong epidemiological evidence linking occupational crystalline silica exposure to silicosis, lung cancer, pulmonary tuberculosis, and chronic obstructive pulmonary disease (COPD) in humans (IARC 2012; WHO 2000). On the contrary, the respiratory health of workers upon inhalation of pyrogenic SAS was shown not to be affected in six epidemiological studies (Antoniou et al. 2023). This difference in effects induced by quartz and pyrogenic silica is supported by data from rodent inhalation studies. Table 3 shows a hazard ranking of the four silica particles derived from in vivo data, based on the types of adverse effects and potency. Inhalation data is abundant for crystalline and pyrogenic silica particles. Some information is available for wet-process synthetic amorphous silica, including colloidal silica. Data from animal studies using the quartz particles Sikron F300 and Min-U-Sil 5 were included in the quartz group in addition to DQ12 because of their structural similarity. The pyrogenic silicas SAS1 and Cab-O-Sil M5 were included in the pyrogenic silica group in addition to NM-203 because of their similar physico-chemical parameters. The colloidal silicas Levasil 200 and Ludox CL-X were considered similar to Silica-Std in terms of physico-chemical parameters and are therefore included as well. Silane surface functionalized colloidal silica was tested in one 90-day exposure study performed according to OECD TG 413, of which the publicly available data as well as data obtained from personal communication were used for establishing the ranking (Anonymous 2021).

**Table 3:** A qualitative ranking of local toxic potency based on results from in vivo studies in which the four SiO_2_ particles used in the in vitro tests presented in this paper were tested

Study	Particle	Exposure duration and concentrations	Fibrosis	Granulomas (and their persistent presence post-exposure)	PMN in BALF elevated after recovery	LOAEC	NOAEC
1) Quartz							
Muhle et al. (1995)	DQ12	2 years, recovery 6 weeks 1 mg/m^3^	Yes	Not assessed	Not assessed	1 mg/m^3^	< 1 mg/m^3^
Weber et al. (2024)	DQ12	90 days, recovery 1 year, 1 mg/m^3^	Yes	Yes, persistent	Yes	1 mg/m^3^	< 1 mg/m^3^
Reuzel et al. (1991)	Sikron F300	90 days, recovery 1 year 60 mg/m^3^	Yes	Yes, persistent	Not assessed	60 mg/m^3^	< 60 mg/m^3^
Henderson et al. (1995)	Min-U-Sil 5	28 days, recovery 6 months 0.1, 1, 10 mg/m^3^	No	Yes, persistent at 10 mg/m^3^	Yes, for 10 mg/m^3^	1 mg/m^3^	0.1 mg/m^3^
Arts et al. (2007)	Min-U-Sil 5	5 days, recovery 3 months 25 mg/m^3^	No	No	Yes	25 mg/m^3^	< 25 mg/m^3^
2) Pyrogenic silica							
Reuzel et al. (1991) Weber et al. (2018) ECHA (2019)	Aerosil 200	90 days, recovery 1 year 1, 6, 30 mg/m^3^	Debated*	Yes, transient	Not assessed	1 mg/m^3^	< 1 mg/m^3^
Weber et al. (2024)	SAS1	90 days, recovery 1 year 0.5, 1, 2.5, 5 mg/m^3^	No	Yes, persistent, but low incidence and low severity	No	2.5 mg/m^3^	1 mg/m^3^
Arts et al. (2007)	Cab-O-Sil M5	5 days, recovery 3 months 1, 5, 25 mg/m^3^	No	No	No	5 mg/m^3^	1 mg/m^3^
3) Colloidal silica							
Anonymous (2021)	CS30-236	90 days, recovery 90 days 25 mg/m^3^	No	Yes, transient	Yes, but only slightly elevated	25 mg/m^3^	< 25 mg/m^3^
Lee and Kelly (1992) Warheit et al. (1991)	Ludox (CL-X)	28 days, recovery 90 days 10, 50, 150 mg/m^3^	No	No	Yes, for 150 mg/m^3^	50 mg/m^3^	10 mg/m^3^
Landsiedel et al. (2014a)	Levasil 200	5 days, recovery 21 days 0.5, 2.5, 10, 50 mg/m^3^	No	No	Yes, for 10 and 50 mg/m^3^	10 mg/m^3^	2.5 mg/m^3^
4) Silane surface functionalized silica							
Anonymous (2021)	CC301	90 days, recovery 90 days 1, 5, 25 mg/m^3^	No	No	Yes, but only slightly elevated for 5 mg/m^3^	5 mg/m^3^	1 mg/m^3^
Landsiedel et al. (2014a)	Silica surface modified**	5 days, recovery 21 days 2, 10, 50 mg/m^3^	No	No	No	> 50 mg/m^3^	50 mg/m^3^

Rank number 1 means most hazardous based on the in vivo studies, followed by ranks 2, 3, and 4. In all inhalation studies, animals were exposed 6 h/day, 5 days/week

*NOAEC* no-observed-adverse-effect concentration, *LOAEC* lowest-observed-adverse-effect concentration, *PMN* polymorphonuclear cells, *BALF* Bronchioalveolar lavage fluid

^*^See Supplementary Table S2 for further information, **modified with polyethyleneglycol, phosphate, or amino groups

The data that were used for the ranking were the histopathological observations fibrosis, fibrogenesis, and granuloma-like lesions, which are considered adverse effects (for the latter depending on incidence, severity, and reversibility, i.e. whether the presence of granulomas was maintained during an exposure free period following the exposure (recovery period)). Additionally, polymorphonuclear (PMN) cells/granulocytes/neutrophils in bronchioalveolar lavage fluid (BALF) were evaluated which can be considered adverse when persistent or progressive after the recovery period. Finally, the concentration of onset of PMN influx and histopathological changes was evaluated to determine the potency of the particle to induce local pulmonary inflammatory effects (lowest-observed-adverse-effect concentration, LOAEC) as well as the concentration at which no adverse effects were noted (no-observed-adverse-effect concentration, NOAEC). The details of the rodent inhalation studies used for this ranking are provided in Supplementary Table S2.

Quartz particles induced the most severe and irreversible effects, and at the lowest exposure concentrations (Table 3). Also, effects increased with time during the exposure-free period (Reuzel et al. 1991). Pyrogenic silica-induced effects were also rather severe, yet mostly reversible, and the NOAEC and LOAEC reported in literature were slightly higher than those reported for quartz in studies where multiple concentrations were used. Colloidal silica particle-induced effects were mild and transient and occurred at higher exposure concentrations. No notable effects were observed for Silica-Silane and other surface modified colloidal silica particles. In meta analyses of the effects of silica particles upon inhalation in rodents it was also found that the potency of crystalline silica is higher compared to amorphous silica (Hadrup et al. 2023; Romeo et al. 2022).

### Comparisons

**Fig. 10:**
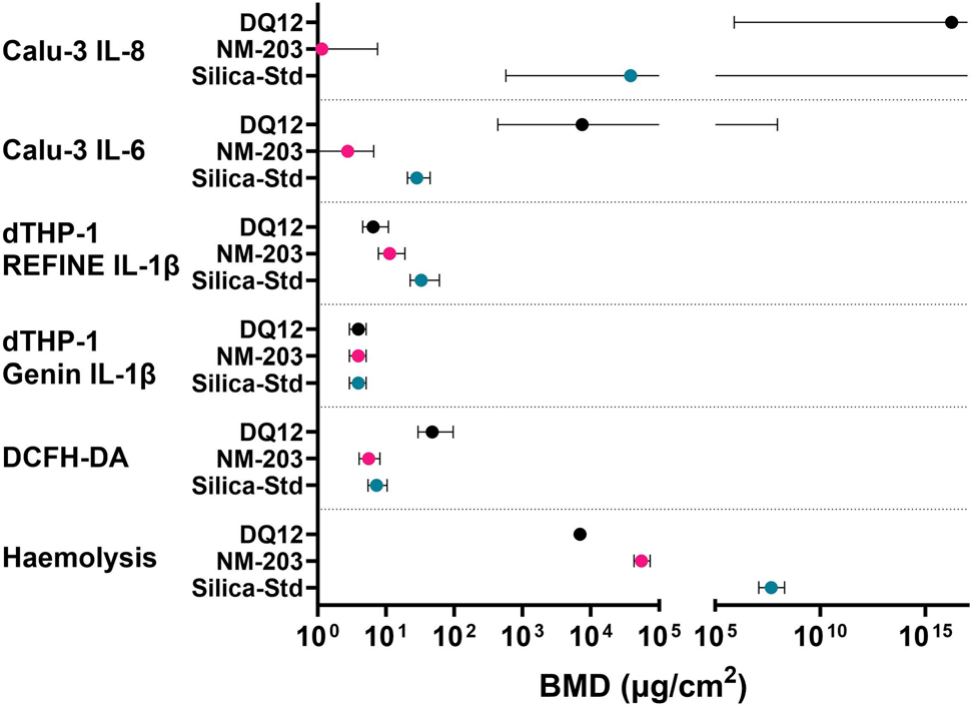
Summary of all hazard potency data obtained for the silica particles in the submerged functional assays, expressed as estimated benchmark dose (BMD) for a 50% increase for each specified marker, with lower confidence interval limit (BMDL) and upper confidence interval limit (BMDU) visualized. Only the results of the assays in which DQ12, NM-203, and Silica-Std were tested, and where effects were observed, are shown. Infinite BMDUs extend off the right side of the x-axis. The haemolysis assay results are shown as BMD (µg/mL) since this assay is carried out in suspension and deposited doses are not applicable

**Fig. 11:**
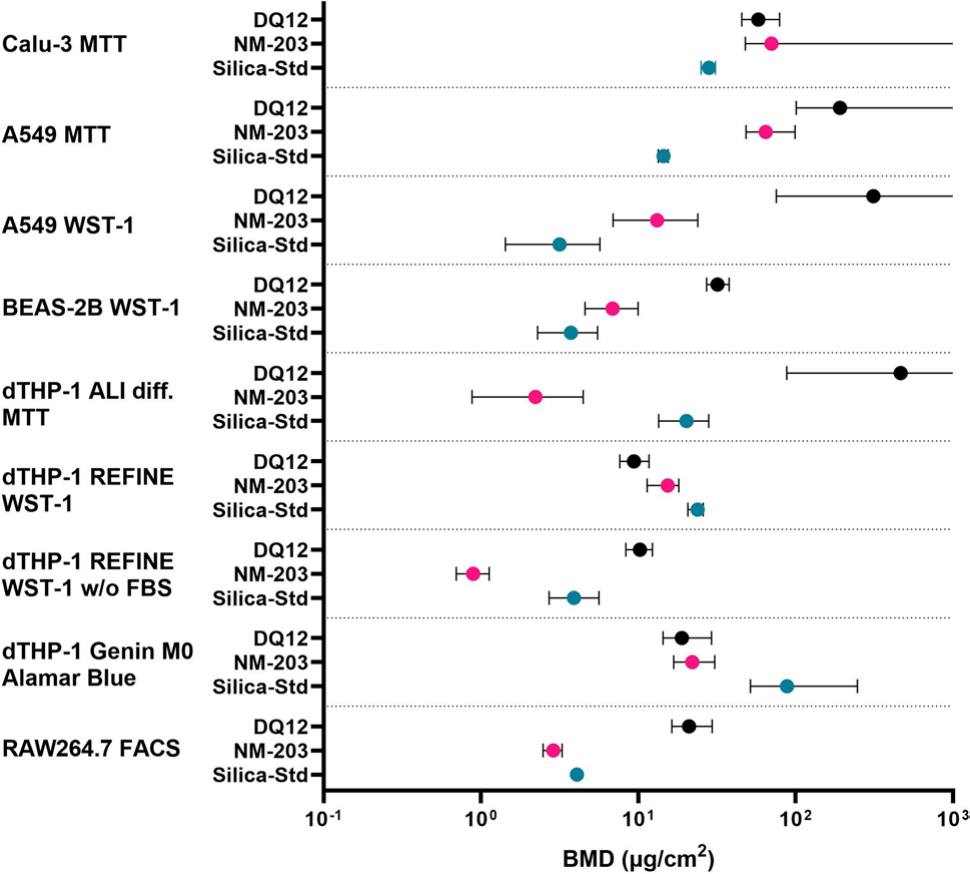
Summary of all hazard potency data obtained for the silica particles in the submerged viability assays, expressed as estimated benchmark dose (BMD) for a 50% reduction in cell viability with lower confidence interval limit (BMDL) and upper confidence interval limit (BMDU) visualized. Only the results of the assays in which DQ12, NM-203, and Silica-Std were tested, and where effects were observed are shown. Infinite BMDUs extend off the right side of the x-axis

Estimated benchmark doses (BMD) and BMD confidence intervals (lower (BMDL) and upper (BMDU) limits) of the effects induced by SiO_2_ particles in all functional assays (cytokine release, ROS production and haemolysis) for which dose–response data were obtained are visualized in Fig. 10 (all BMDLs and BMDUs can be found in Supplementary Table S3). Figure 11 is similar but for all viability data (all BMDLs and BMDUs can be found in Supplementary Table S4). Administered doses (µg/mL) were corrected for deposition efficiency (Table 2) to obtain deposited doses (µg/cm^2^) which were used for BMD modelling. There is no clear relation between particle type and potency in the functional assays performed (Fig. 10). In the viability assays, Silica-Std and NM-203 are more often found on the left side of the graph (lower BMD, higher potency), and DQ12 more often on the right side of the graph (Fig. 11), which does not correspond to the in vivo hazard ranking.

Table 4 shows a summary of all hazard potency rankings obtained in all assays. For establishing the rankings, the overlap between BMD confidence intervals (BMDL and BMDU) was assessed within each functional assay (Supplementary Table S3) and viability assay (Supplementary Table S4) for which dose–response data was obtained. When BMD CIs between two or more particles overlap, they are considered to not significantly differ in their potency and are assigned the same rank. Some assays were performed at one dose, which is not preferred for establishing hazard rankings. For one-dose data (Supplementary Table S5) the 95% CI around the magnitude of the response is used for establishing the ranking. For qPCR data, this is depicted in fold change of gene expression. For FRAS data this is depicted in biological oxidative damage (BOD) in µM trolox equivalent units (TEU). The hazard rankings were compared to the ranking based on in vivo data: DQ12 > NM-203 > Silica-Std > Silica-Silane (Table 3).

**Table 4:**
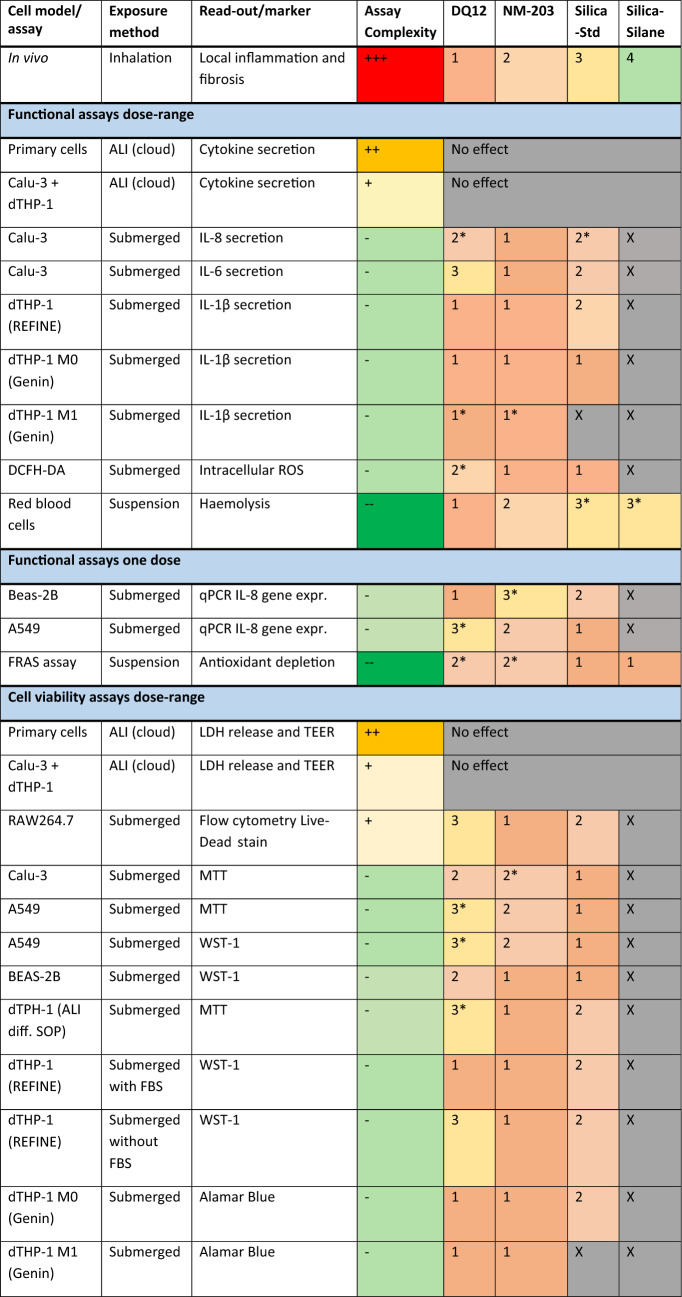
Summary of all qualitative rankings obtained in all assays

^*^no statistically significant effect in the assay. The lowest rank is assigned when the particle did not induce a statistically significant effect. 1 (amber)  = most potent, followed by 2 (light amber), 3 (light yellow), and 4 (light green). Particles receive the same rank when BMD confidence intervals overlap. For one-dose data, the amplitude of the effect is used for establishing the ranking. Rankings start at 1. X = not assessed in the assay. Assay complexity is depicted on an arbitrary scale from + + + (red) to − − − (green), with + + + being the most complex and − − − the least complex

It can be noted that rankings depend on assay, cell type, and read-out used. From the functional assays, the ALI models showed no effect at the doses tested, two submerged models ranked DQ12 the least potent (Calu-3 IL-6 secretion and A549 IL-8 gene expression), and one submerged model could not distinguish between neither particle types (dTHP-1 M0 Genin et al. (2015) protocol). The REFINE SOP for IL-1β secretion showed a good distinction between DQ12 and NM-203 on the one hand and Silica-Std on the other hand. The RBC haemolysis assay was the only assay that could accurately rank DQ12, NM-203, and Silica-Std, but it could not distinguish between Silica-Std and Silica-Silane.

dTHP-1 cell viability as tested following the REFINE SOP showed the same ranking as its IL-1β release, to which the Genin et al. (2015) M0 protocol viability results also corresponded. All other rankings in the viability assays did not correspond to the in vivo toxicity ranking. For viability data it should also be noted that the inclusion of FBS had an influence on test outcomes by affecting deposition efficiency. The rankings within each assay at administered doses versus the ranking at deposited doses were however not affected, indicating that this effect was not large. However, testing without FBS did change the ranking in cell viability in the dTHP-1 model using the REFINE SOP (NM-203 > Silica-Std > DQ12) as compared to testing following the original SOP with FBS supplementation (DQ12 = NM-203 > Silica-Std), both when the rankings were based on administered and deposited doses.

The dissolution half times of the four particles in phagolysosomal fluid were obtained from literature (depicted in Table 1) and are as follows: 3.6 days for Silica-Silane, 3.9 days for Silica-Std, 35 days for NM-203, and an estimated > 100 days for DQ12. These dissolution half times correspond to the in vivo hazard ranking, although overlap between confidence intervals between these values could not be excluded.

## Discussion

For the purpose of pre-regulatory hazard screening in the context of SSbD, it is desirable that assays have the capacity to correctly rank NMs, prioritize safe alternatives, and flag NMs of concern. To this end, in vitro hazard rankings of silica particles were compared to a hazard ranking derived from in vivo data. The assays were selected based on the (expected) MOA towards pulmonary inflammation and fibrosis. Twenty-four assays consisting of different combinations of cellular and acellular models, read-outs, and exposure conditions (e.g. different cell types, submerged vs. air–liquid interface exposure) were carried out of which twelve functional (cytokine secretion, oxidative stress, and haemolysis), and twelve related to cell viability. Data on dissolution rates of the silica particles were considered as well. The particle of most concern in this current paper is DQ12 due to its ability to induce silicosis, which is characterized by persistent inflammation and fibrosis (IARC 2012; WHO 2000).

### Particle hazard ranking

#### In vitro hazard ranking

The hazard ranking obtained using the RBC haemolysis assay corresponded best with the in vivo hazard ranking, with crystalline and pyrogenic silica inducing a strong effect which was still distinguishable in terms of potency. However, the response to each colloidal silica was negligible, which was not in line with the ranking based on in vivo data. The haemolysis assay has previously been used to demonstrate the surface reactivity of crystalline silica (Clouter et al. 2001; Driscoll 1995; Duffin et al. 2001; Geh et al. 2005; Nolan et al. 1981). The test has also been shown to be useful in the detection of membranolytic effects induced by a number of other types of particles and fibres, including metal oxides, polystyrene, asbestos, and refractory ceramic fibres (Cho et al. 2013; Koshi et al. 1968; Lu et al. 2009; Luoto et al. 1997; Mc Guinnes et al. 2010), and was previously shown to correlate well with in vivo inflammation in response to metal oxides (Cho et al. 2013; Lu et al. 2009), and crystalline silica (Clouter et al. 2001; Duffin et al. 2001). Here we have confirmed the correlation of the haemolysis assay with pulmonary inflammation induced by crystalline silica, and further demonstrated this to be true for pyrogenic silica.

The hazard ranking obtained by assessing IL-1β secretion by M0 dTHP-1 cells following the REFINE SOP (Vandebriel et al. 2022) also corresponded well with the in vivo hazard ranking, where a strong but non-distinguishable effect of crystalline and pyrogenic silica, and a lower but significant effect of Silica-Std were observed. IL-1β secretion from peritoneal macrophages has previously been shown to correlate well with haemolysis in response to crystalline and vitreous silica fibres (Pavan et al. 2014), which we also demonstrate here for crystalline and pyrogenic silica using dTHP-1 macrophages. However, in the present work no distinction could be made between DQ12 and NM-203 using dTHP-1 cells. The method of THP-1 differentiation and polarization had a large impact on in vitro hazard ranking. No distinction could be made in particle potency when monocyte differentiation to M0 was carried out following the methods published in Genin et al. (2015). The experiments using dTHP-1 cells were however carried out in different labs and interlaboratory variability may have played a role here. dTHP-1 cells were previously shown to demonstrate considerable interlaboratory variation in response to LPS and particle exposures (Piret et al. 2017; Xia et al. 2013). The hazard ranking in cell viability also differed between differentiation protocols and between FBS and non-FBS experiments following the REFINE protocol. The difference in effects observed upon THP-1 differentiation following different protocols highlights the importance of protocol standardization. The REFINE SOP, which has already undergone standardization (Vandebriel et al. 2022) and showed the most accurate ranking, is therefore the preferred choice to test for NLRP3 inflammasome activation by silica particles.

McLean et al. (2023) had found secretion of both IL-1β and IL-6 by simple submerged macrophage models to be promising in vitro screening tools for pulmonary fibrosis. Here, IL-6 in M0 and M1 dTHP-1 macrophages was found not to be a suitable indicator of silica pulmonary toxicity as no response was observed upon exposure to the silica particles. The Calu-3 bronchial epithelial cells did secrete IL-6 in response to silica particles, but the resulting hazard ranking did not match the in vivo hazard ranking.

The cell types other than dTHP-1 macrophages (A549, Calu-3, BEAS-2B, RAW264.7) and other pro-inflammatory cytokines assessed (either by ELISA or qPCR) generally demonstrated a low correlation to the in vivo ranking of the four silicas. DQ12 is commonly used as a positive control for in vitro pro-inflammatory responses, but its suitability was already questioned previously in a study where it was found that the responses to DQ12 depended on the cell type used and on whether the particles had been reactivated by fractioning (Meldrum et al. 2022). More pronounced effects were expected in the current study given the haemolytic potential demonstrated by the DQ12 particles.

Cell viability of lung epithelial cell lines (A549, Calu-3, and BEAS-2B) also proved to be an unsuitable indicator of the in vivo hazard ranking as DQ12 was regularly shown to have the lowest cytotoxic potency.

#### Hazard ranking based on data derived from in vivo studies

The comparison of acute in vitro effects to outcomes of inhalation studies introduces several uncertainties. Firstly, little information is published about the acute adverse effects of inhalation of silica particles in rodents, as most studies are sub-chronic (90 days) studies as is performed for regulatory purposes and more relevant for human occupational exposures (Landsiedel et al. 2014b). This makes the current comparison (24 h in vitro vs 90 days in vivo) a large step, and comparisons of our data to acute in vivo toxicity data could help identify the source of the discrepancies between the hazard rankings. The shortest inhalation study where two of the case study materials were tested was a 5 day inhalation study, in which the effects of crystalline silica were less severe as compared to pyrogenic silica in terms of cytotoxicity biomarkers in BALF and histopathological changes after the exposure period, yet more severe at the 3 month recovery timepoint (Arts et al. 2007), indicating that the adverse effects of crystalline silica require more time to develop. This likely explains why DQ12 was not consistently ranked having the highest potency in the in vitro assays and raises the question whether 24 h in vitro experiments alone are informative enough for pre-regulatory screening of crystalline silica particles and similar particles with a very slow dissolution rate that might accumulate in the lungs. Secondly, the comparison of human cell types in vitro to adverse outcomes from rodent studies may introduce uncertainty. Lung morphology, particle deposition patterns, and clearance responses differ substantially between humans and rats (Bergmann 1998; Brown et al. 2005), and a comparison of human based in vitro assay results to human data is therefore preferred. Fortunately, the human epidemiological data support the hazard ranking between quartz and pyrogenic silica particles, but human data on the other two silicas from the present study are missing to draw a firm conclusion. Thirdly, the in vivo ranking in pulmonary toxicity used in this study is based on the type of observation (fibrosis, granuloma), reversibility, and potency of pulmonary inflammation. The in vitro responses are ranked according to potency alone. Reversibility is crucial in silica toxicity in vivo but could not be assessed in vitro due to the limited assay durations, as discussed further below*.* Fourthly, the effect of the exposure method might introduce uncertainties as well. The characteristics of the aerosols generated for the in vivo studies may differ greatly from the characteristics of the particles present in CCM, as do their protein coronas. Furthermore, particle characteristics may differ between CCM types, and in assays where other media such as HBS (FRAS assay), or PBS (haemolysis assay) are used. To avoid creating a protein corona with a composition with little relevance for inhalation exposures, the use of FBS was omitted as much as possible in the in vitro exposures in this study. However, a few of the THP-1 protocols required FBS, and it has previously been established that the presence of serum reduces the interaction of silica particles with cell membranes and reduces their uptake (Lesniak et al. 2012). The sonication procedures also differed between SOPs. Finally, the in vivo ranking contains a certain degree of (unknown) uncertainty related to the differences in rat strains, exposure concentrations, mass median aerodynamic diameter (MMAD), pathologists who graded the slides, etc*.* between studies. Therefore, we cannot be certain that the in vivo hazard potency confidence intervals are not overlapping between particles.

### Considerations of lung burden and persistence of inflammation for in vitro screening

The higher toxic potential of quartz particles as compared to pyrogenic silica is partly associated with their retention in lung tissue (Arts et al. 2007). Since DQ12 was not consistently ranked as most potent in the in vitro assay outcomes, the adverse outcome (i.e. severe and irreversible pulmonary inflammation) of exposure to DQ12 in vivo is likely to be strongly linked to continued presence of particle in the lung as well as to surface-related effects. Weber et al. (2024) found that for pyrogenic silica particles, the severity and reversibility of effects upon inhalation in rats were also largely dependent on particle solubility. Persistency of effects is not assessed in acute in vitro assays as the experiments are not of long enough duration. More complex in vitro experiments using longer exposures as well as a recovery period are not necessarily preferable for a pre-regulatory hazard screening approach due to the added complexity, but might be required for increased certainty when testing poorly soluble particles. In this study we found Calu-3 cells unsuitable for long-term exposures due to observed overgrowth and cell death after seven days at the ALI, which was in contrast with previously published reports in He et al. (2021) and García-Rodríguez et al. (2024).

Clearance of particles from the lungs is amongst others affected by their dissolution rate and uptake and clearance by macrophages (Geiser 2010). The dissolution rates of the four silica particles tested in this study correspond to the in vivo hazard ranking. Therefore, a combination of a dissolution assay combined with two or more mechanism specific assays may be the key to predicting silica-induced adverse effects in a pre-regulatory context. Both slow dissolution as well as very quick dissolution may serve as a warning in pre-regulatory hazard screening, as slow dissolution may point to persistency, and quick dissolution may point to a possible shedding of toxic ions. The challenges of simulating biological fluids for in vitro dissolution studies (Innes et al. 2021) and progress towards standardisation of acellular dissolution methodology (Zanoni et al. 2022) have recently been described, and an approach combining dissolution rate with particle reactivity and pro-inflammatory responses using similarity assessment was previously proposed for grouping of NMs (Braakhuis et al. 2021). Here we confirm that such an approach is suitable for pre-regulatory screening of silica particles in the context of SSbD. According to our results, it might be necessary to assign a larger weight to the dissolution data during data interpretation.

### Detecting SiO_2_ effects in vitro

#### Mechanism of action

The activation of the NLRP3 inflammasome is a well-known MOA of crystalline SiO_2_ particles, which results in pro-inflammatory responses and pyroptosis, a form of cell death associated with clearance of intracellular pathogens characterized by lysis and cell swelling (Croissant et al. 2020; Hornung et al. 2008; Schroder & Tschopp 2010). Additionally, the activation of the NLRP3 inflammasome has been linked to the transition from acute to chronic inflammation (Cox et al. 2020). Therefore, IL-1β secretion, through NLRP3 inflammasome activation serves as a good in vitro indicator for silica toxicity. To avoid the misinterpretation of non-NLRP3 specific IL-1β release, NLRP3 inflammasome activation should be confirmed using NLRP3 deficient THP-1 cells (Vandebriel et al. 2022). Haemolysis and NLRP3 inflammasome activation are likely both induced through a membrane-specific particle-membrane interaction, with the red blood cell and with endosomes/lysosomes respectively (Pavan et al. 2020), explaining why the haemolysis assay was also found as a potentially good marker for silica toxicity in this study. In literature, NLRP3 inflammasome activation is seen for crystalline and pyrogenic silica, but not for colloidal silica particles (Croissant et al. 2020; Zhang et al. 2012). In the present study, exposure to (colloidal) Silica-Std induced IL-1β secretion but not haemolysis (tested up to 15 mg/mL). This could mean that IL-1β secretion is a more sensitive marker than haemolysis or that IL-1β was secreted through another MOA, which could be clarified by testing with NLRP3 deficient THP-1 cells.

#### ROS

Although often described as important events in silica particle toxicity (Napierska et al. 2010), the induction of free radicals (measured in the FRAS assay) and intracellular ROS, produced either by the particles or by the cell in response to the particles (measured in the DCFH assay), did not correlate to the hazard ranking based on in vivo data. NM-203 did not induce an effect in the FRAS assay but did induce an effect in the DCFH assay. Unstable structures that form on the pyrogenic silica (NM-203) surface upon heating, such as three-membered rings, are reservoirs for ROS generation (Croissant et al. 2020; Zhang et al. 2012). An effect of pyrogenic silica in the FRAS assay would therefore be expected but was not observed (tested up to 40 mg/mL). This could indicate lower assay sensitivity of the FRAS assay as compared to the cellular DCFH assay. Colloidal silicas, which are produced at low temperatures, are not expected to induce ROS (Croissant et al. 2020; Zhang et al. 2012). Silica-Std did however induce an effect in the FRAS and DCFH assay, indicating ROS production and possible oxidative stress. DQ12 did not induce an effect in the FRAS and DCFH assay. Nearly-free silanols, the surface structures responsible for most of crystalline silica toxicity are indeed membranolytic (reflected in the haemolysis assay), but do not induce particle-derived ROS (Pavan et al. 2020). Overall, cellular and acellular ROS production are likely not good indicators of pulmonary inflammation by silica particles, but only a small selection of assays was assessed here.

### Relevance of findings for SSbD hazard screening

Evaluation of available adverse outcome pathways (AOPs) can identify mechanisms and overlapping key events (KEs) which support prioritization of endpoints for testing (Halappanavar et al. 2020). It has to be noted that all endpoints assessed in this study (secretion of pro-inflammatory cytokines, ROS production, and cellular toxicity) are early KEs in the existing AOPs leading to adverse pulmonary effects (Halappanavar et al. 2020). These in vitro effects are also not necessarily considered adverse if they were to occur in vivo*,* since a certain degree of (transient) pro-inflammatory responses, such as the recruitment of PMN cells through the secretion of cytokines, is necessary for the clearance of particles from the lungs (Weber et al. 2024). In fact, pro-inflammatory markers were even questioned as a suitable marker for persistent inflammation in in vivo regulatory studies (Poland et al. 2024). The activation of the NLRP3 inflammasome is also considered beneficial for the clearance of foreign substances by recruiting neutrophils through IL-1β (Sun et al. 2013). Assessing later cellular level KEs might increase the accuracy of the predictions but reduces the simplicity of the screening approach.

In AOP 173 (substance interaction with the pulmonary resident cell membrane components leading to pulmonary fibrosis (Halappanavar et al. 2023)) the KE ‘secretion of pro-inflammatory cytokines’ is rather broad and therefore difficult to translate into an in vitro assay. Here we show that not all cytokines and cell types prove to be good markers for pulmonary inflammation and fibrosis, and suggest IL-1β secretion by macrophages and haemolysis as potential markers for silica-induced pulmonary toxicity. We recommend confirming their suitability with other particles following a similar MOA (such as TiO_2_ NMs, which have also been shown to activate the NLRP3 inflammasome (Sun et al. 2013)), and determining suitable in vitro markers for particles with a different MOA (such as ZnO). This together with in silico screening approaches may serve as a basis for a SSbD hazard screening approach. Furthermore, IL-1β secretion by M0 macrophages and the haemolysis assay have the potential to be included into an integrated approach to testing and assessment (IATA) for the assessment of pulmonary inflammation using NAMs in a regulatory context. However, validation in terms of material applicability domain, predictivity, and inter-laboratory reproducibility is required first.

### Air liquid interface versus submerged conditions

Culturing and exposing cells at the ALI more closely resembles the physiological situation in the lungs as compared to submerged exposures (Paur et al. 2011). However, no increased predictivity of ALI exposures over submerged exposures was found in previous research (Di Ianni et al. 2022; McLean et al. 2023). Additionally, the use of ALI models was shown to introduce high amounts of interlaboratory variability (Braakhuis et al. 2023; Petersen et al. 2021). ALI exposures are however useful to test particles which are buoyant or very slowly settling resulting in a low dose-rate in a submerged setting, such as Silica-Silane in the present study. Exposing cells at the ALI using a cloud system and a QCM, as performed in this study, has the advantage of an easy assessment of the deposited dose, which is more complicated for submerged culturing conditions (Bannuscher et al. 2022; Ding et al. 2020). In the present study, it was found that simple submerged models outperform ALI models for the detection of the effects of silica particles, which is in line with previous research (Di Ianni et al. 2022).

The Calu-3 + dTHP-1 co-culture model showed no response to particles at the ALI in a previous (interlaboratory) study, which the authors suggested was due to doses that might have been too low (Braakhuis et al. 2023). The deposited doses used in the present study were higher than those used in Braakhuis et al. (2023), but might still have been too low to capture any effect after 24 h of exposure. The lowest dose tested on the Calu-3 bronchial epithelial cells in a submerged exposure in this study was 25 µg/mL, and at this dose IL-8 and IL-6 were secreted in response to NM-203 exposure. This dose corresponds to 8.6 µg/cm^2^ (considering 0.2 mL volume in the well, 0.32 cm^2^ surface area of the well, and a deposition efficiency of 55%), which exceeds the highest dose tested at the ALI, which was 4.2 µg/cm^2^. dTHP-1 macrophage responses submerged and at the ALI are difficult to compare as different cytokines were assessed, and differentiation protocols and cell seeding densities were not aligned between (previously established) protocols. The BMDL of IL-1β secretion following the Genin et al. (2015) M0 differentiation protocol was 2.92 µg/cm^2^ for NM-203 after 24 h of exposure, using a cell density ± threefold lower than the dTHP-1 cell density in the ALI co-culture. Therefore, a response at the ALI would be expected, but was not observed. It must be noted that IL-1β was not assessed in the ALI experiments. M0 differentiation following the same protocol that was used for ALI exposures, but exposing submerged, resulted in a BMDL of 0.88 µg/cm^2^ for cell viability. No cytotoxicity was observed at the ALI, meaning that cells at the ALI might be less responsive due to their direct exposure to the air, and limited excess to cell culture medium. The experiments were however carried out in different labs and interlaboratory variations between dTHP-1 cells have been observed previously (Piret et al. 2017; Xia et al. 2013). For the purpose of pre-regulatory hazard ranking in the context of an SSbD approach, we recommend to further increase the doses used in ALI experiments to allow for comparative ranking and prioritization.

## Conclusions

The ranking of four silica particles based on in vitro results depended on the cell type, read-out, and the exposure method used. We conclude that the haemolysis assay and IL-1β secretion by M0 dTHP-1 macrophages are promising in vitro indicators for silica pulmonary toxicity in a pre-regulatory hazard screening approach because of their ability to identify DQ12 as the particle of most concern. Further research is needed to assess the applicability of these assays for other NMs known to induce pulmonary inflammation. Furthermore, we stress the importance of determining deposited dose of all particles when performing in vitro hazard screening, especially when establishing hazard rankings.

We also acknowledge the numerous endpoints and cell types that did not correlate with silica pulmonary toxicity which demonstrates, in general, the limited capacity of 24 h in vitro exposures to estimate adverse effects in vivo*,* especially of poorly soluble particles. It is plausible that this is caused by the chronic and persistent nature of DQ12-induced adverse effects and therefore an indication of the potential for particle retention (e.g. through assessment of dissolution rate and macrophage uptake), is crucial to strengthen any pre-regulatory hazard screening strategy.

## Supplementary Information

Below is the link to the electronic supplementary material.

Supplementary file1 (PDF 3050 KB)

## Data Availability

The dataset(s) supporting the conclusions of this article will be publicly available in the eNanoMapper database from the 1 st of June 2025.
