# Supplementary material for: Application of in vitro pulmonary models for hazard screening of silica particles
Source: Arch Toxicol. 2025 Jun 25;99(10):4105–30. doi: 10.1007/s00204-025-04100-5 (PMC12454472; doi:10.1007/s00204-025-04100-5)
Supplement: Supplementary file 1 — Supplementary file1 (PDF 3050 KB) [file 204_2025_4100_MOESM1_ESM.pdf]

Supplementary materials: Supporting data

Application of *in vitro* pulmonary models for hazard screening of silica particles

Nienke Ruijter[1], Hedwig Braakhuis[1,2], Alberto Katsumiti[3], Itziar Polanco Garriz[3], Marie Carriere[4], Ilaria Zanoni[5], Ana Candalija[6], Jessica Marshall[7], Jolanda Vermeulen[1], Flemming R. Cassee[1,8,*], Matthew Boyles[7,9]

[1] National Institute for Public Health & the Environment (RIVM), Bilthoven, The Netherlands
[2] TNO, Risk Analysis for Prevention, Innovation and Development, Utrecht, The Netherlands (current affiliation)
[3] GAIKER Technology Centre, Basque Research and Technology Alliance (BRTA), Zamudio, Spain
[4] Univ. Grenoble-Alpes, CEA, CNRS, Grenoble INP, IRIG, SyMMES, CIBEST, Grenoble, France
[5] CNR-ISSMC, National Research Council of Italy, Institute of Science, Technology and Sustainability for Ceramics, Faenza, Italy
[6] Leitat Technological Centre, Barcelona, Spain
[7] Institute of Occupational Medicine (IOM), Edinburgh, UK
[8] Institute for Risk Assessment Sciences (IRAS), Utrecht University, Utrecht, The Netherlands
[9] Centre for Biomedicine and Global Health, School of Applied Sciences, Edinburgh Napier University, Sighthill Campus, Edinburgh, UK

*Corresponding author: Flemming Cassee. Flemming.cassee@rivm.nl

Table S1 Characteristics of silica particles (100 µg/mL) used in this study in different media.

Parameter and medium	DQ12	NM-203	Silica-Std	Silica-Silane
**Water**				
Critical delivered sonication energy (J/mL)	126 ± 31.2	90 ± 31.2	0	0
**NaCl (20 mM) in water**				
Z-average (nm)	514.3 ± 27.4	166.1 ± 2.1	20.8 ± 0.6	20.7 ± 1.0
Polydispersity	0.24 ± 0.04	0.13 ± 0.04	0.18 ± 0.04	0.22 ± 0.02
Zeta-potential (mV)	-42.4 ± 1.8	-21.5 ± 1.4	N/A	N/A
**MEM-Glutamax, no FBS**				
Z-average (nm)	597.7 ± 21.5	169.8 ± 2.4	23.9 ± 1.3	19.8 ± 0.5
Polydispersity	0.16 ± 0.04	0.12 ± 0.5	0.25 ± 0.01	0.20 ± 0.02
Zeta-potential (mV)	-32.6 ± 1.5	-31.8 ± 2.7	N/A	N/A
Effective density (g/cm^3)	1.49	N/A	N/A	N/A
**RPMI-Glutamax, no FBS**				
Z-average (nm)	573.3 ± 11.3	167.8 ± 2.6	22.0 ± 1.9	20.3 ± 0.6
Polydispersity	0.23 ± 0.05	0.17 ± 0.07	0.23 ± 0.02	0.21 ± 0.01
Zeta-potential (mV)	-34.0 ± 2.2	-31.6 ± 2.6	N/A	N/A
Effective density (g/cm^3)	1.43	N/A	N/A	N/A
**RPMI-Glutamax, 10% FBS**				
Z-average (nm)	401.2 ± 23.8	137.3 ± 3.7	74.7 ± 2.1	16.8 ± 0.9
Polydispersity	0.56 ± 0.05	0.50 ± 0.03	0.41 ± 0.05	0.41 ± 0.05
Zeta-potential (mV)	-15.6 ± 1.1	-11.9 ± 1.0	N/A	N/A
Effective density (g/cm^3)	1.32	N/A	N/A	N/A

RPMI and MEM were chosen as they represent media with the most additives and with the least additives respectively. Zeta-potential and effective density could not be obtained for Silica-Std and Silica-Silane as the electrical current likely induced dissolution, and there was no pellet visible after analytical centrifugation. DQ12 and NM-203 were prepared in water by probe sonication with critical delivered sonication energy (1:40 min and 2:20 min at 10% amplitude respectively) before being diluted in medium.

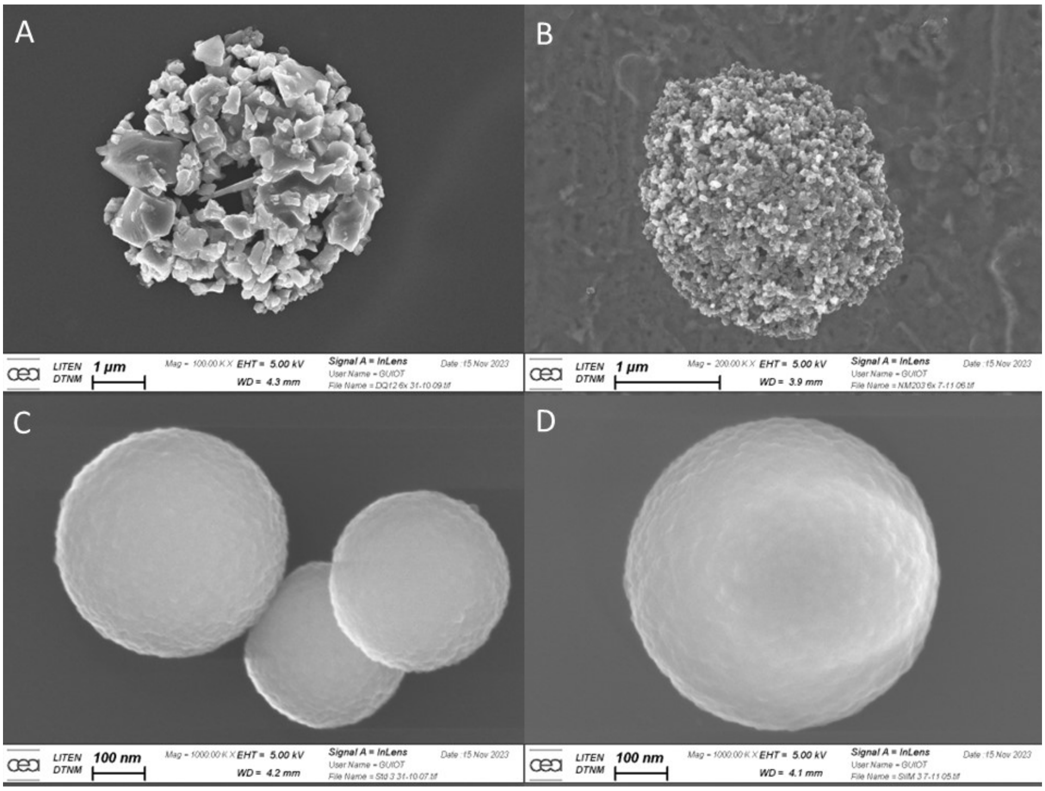

Fig. S1 Scanning Electron Microscopy images of aerosolized and dried DQ12 (A), NM-203 (B), Silica-Std (C), and Silica-Silane (D) following nebulization in the RIVAES, and collecting and drying the aerosol on TEM grids. The spherical organization of the particles is due to the drying of the aerosol droplet that they were in. It can be appreciated that the DQ12 particles are larger and appear more rough than the NM-203 particles.

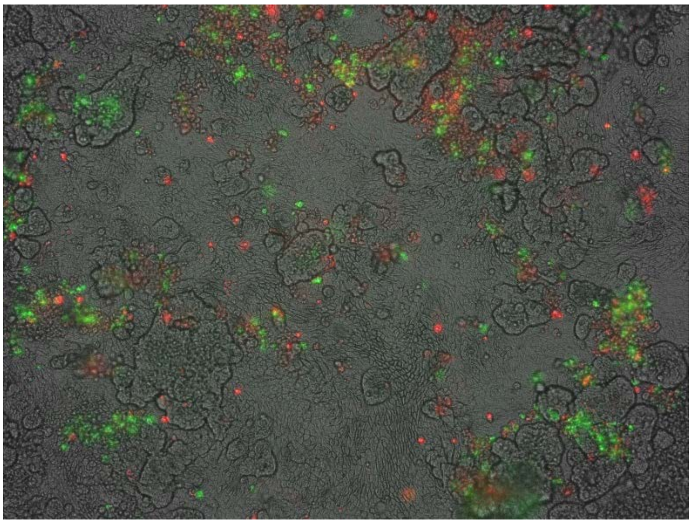

Fig. S2 Fluorescence microscopy picture of Calu-3 (grey) co-culture with dTHP-1 (green) 48 h after creation of co-culture at the ALI. Cell death is stained in red using propidium iodide.

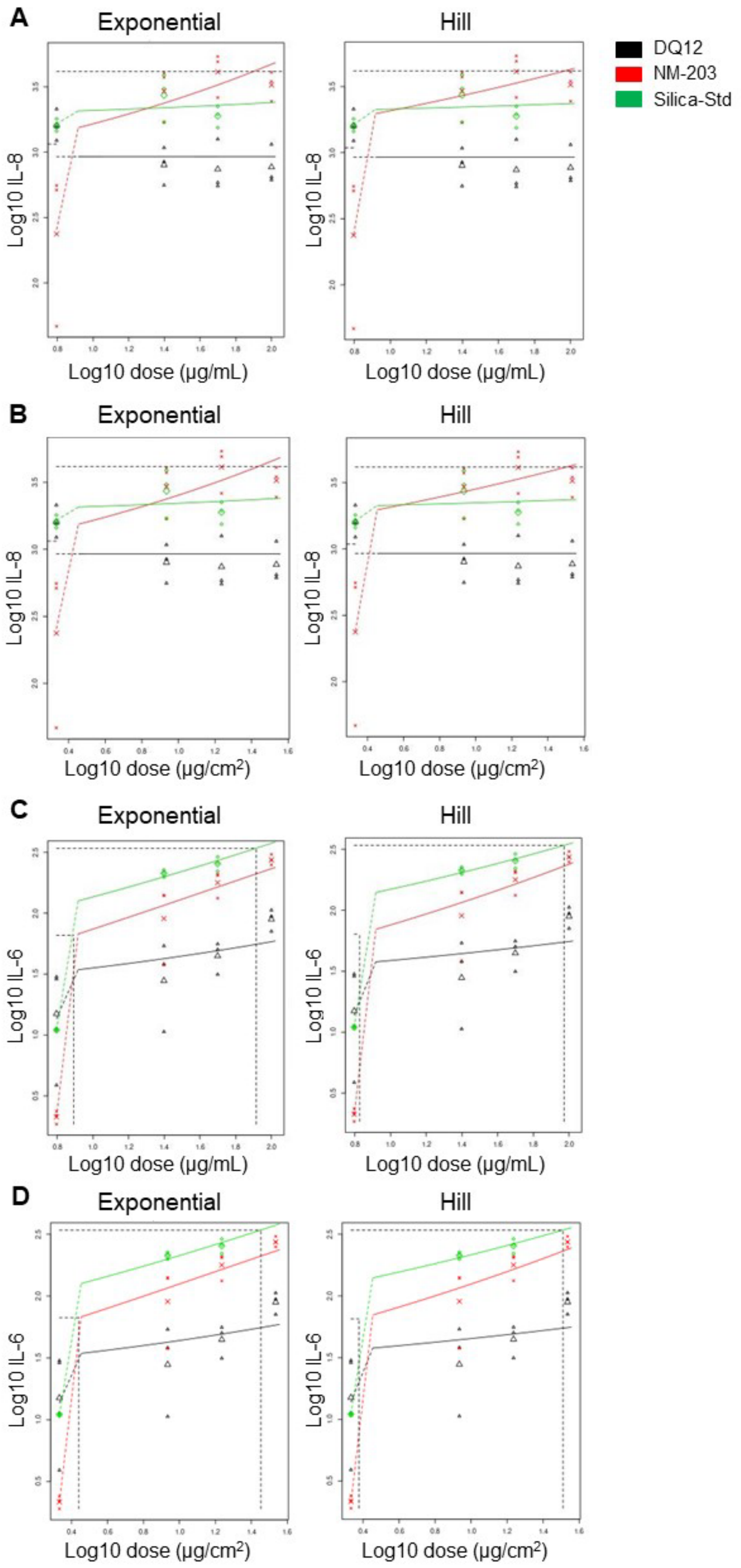

Fig. S3 PROAST dose-response curves of Calu-3 cytokine secretion, supporting Figure 2 in the manuscript. IL-8 secretion (A+B) with CES 350% and IL-6 (C+D) with CES 3000%, as assessed using ELISA. Administered doses (A+C) and deposited doses (B+D). This experiment was carried out without FBS supplementation.

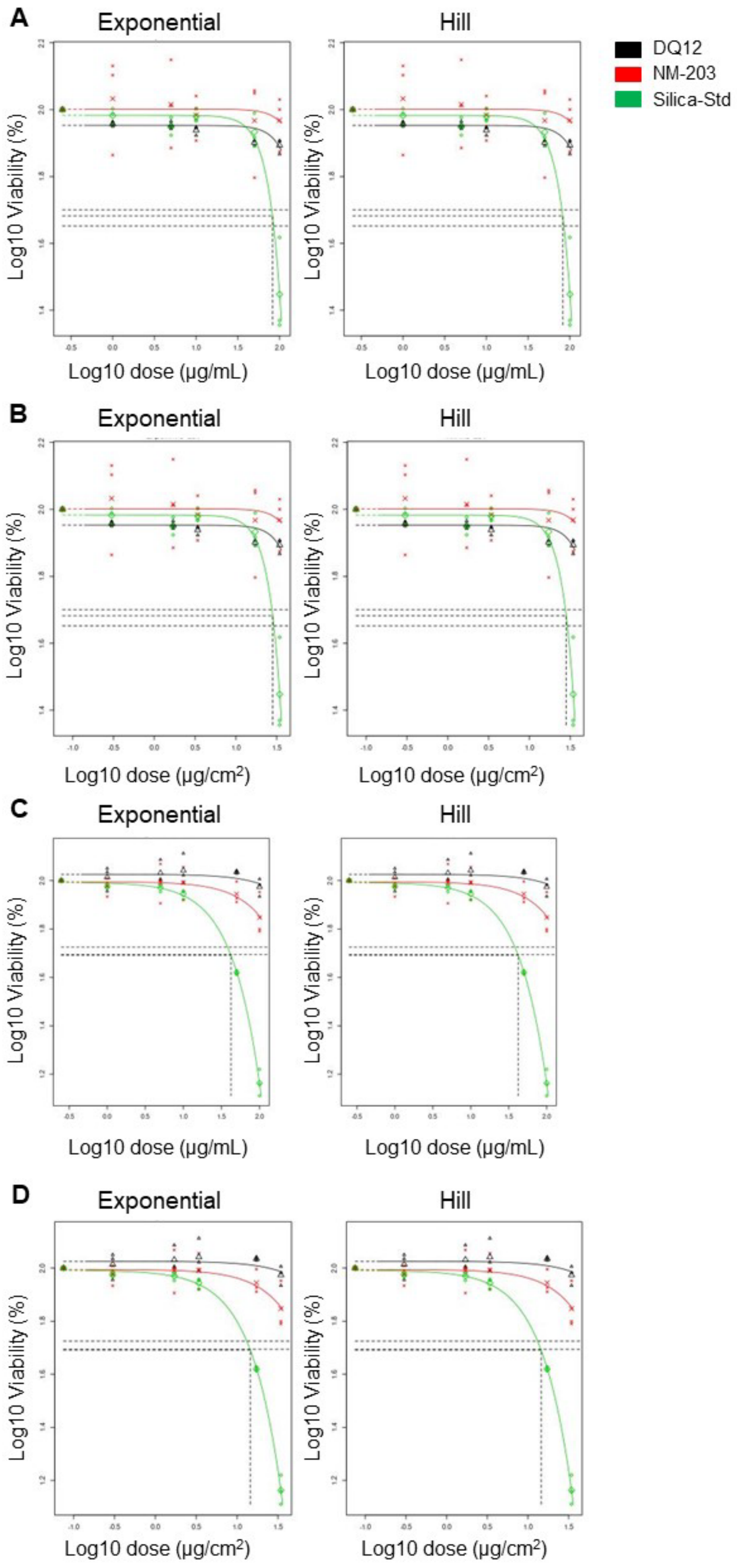

Fig. S4 PROAST dose-response curves of Calu-3 viability (A+B) and A549 viability (C+D) assessed using MTT assay, supporting Figure 2 in the manuscript. Administered doses (A+C) and deposited doses (B+D). CES = 50%. This experiment was carried out without FBS supplementation.

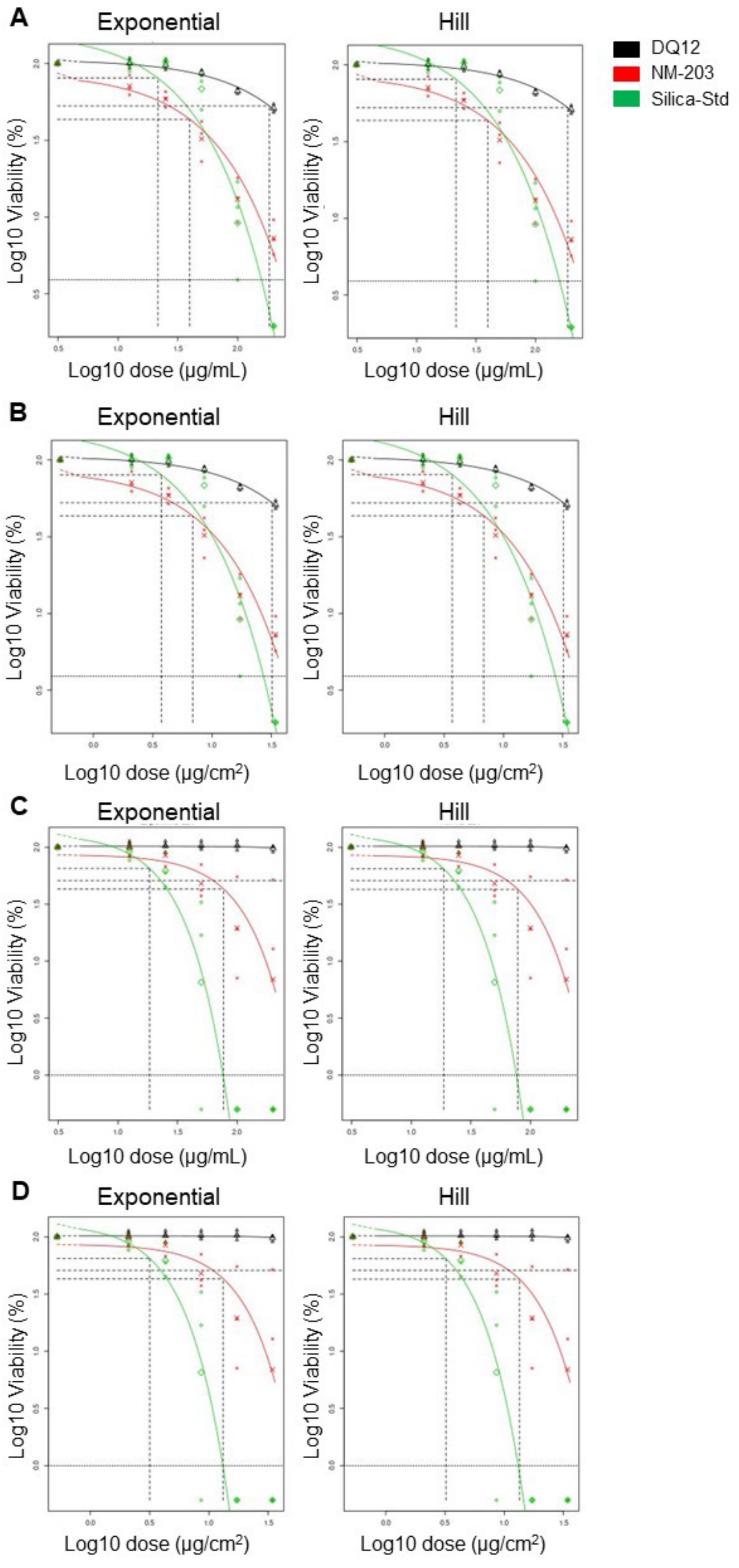

Fig. S5 PROAST dose-response curves of BEAS-2B viability (A+B) and A549 viability (C+D) assessed using WST-1 assay, supporting Figure 3 in the manuscript. Administered doses (A+C) and deposited doses (B+D). CES = 50%. This experiment was carried out without FBS supplementation.

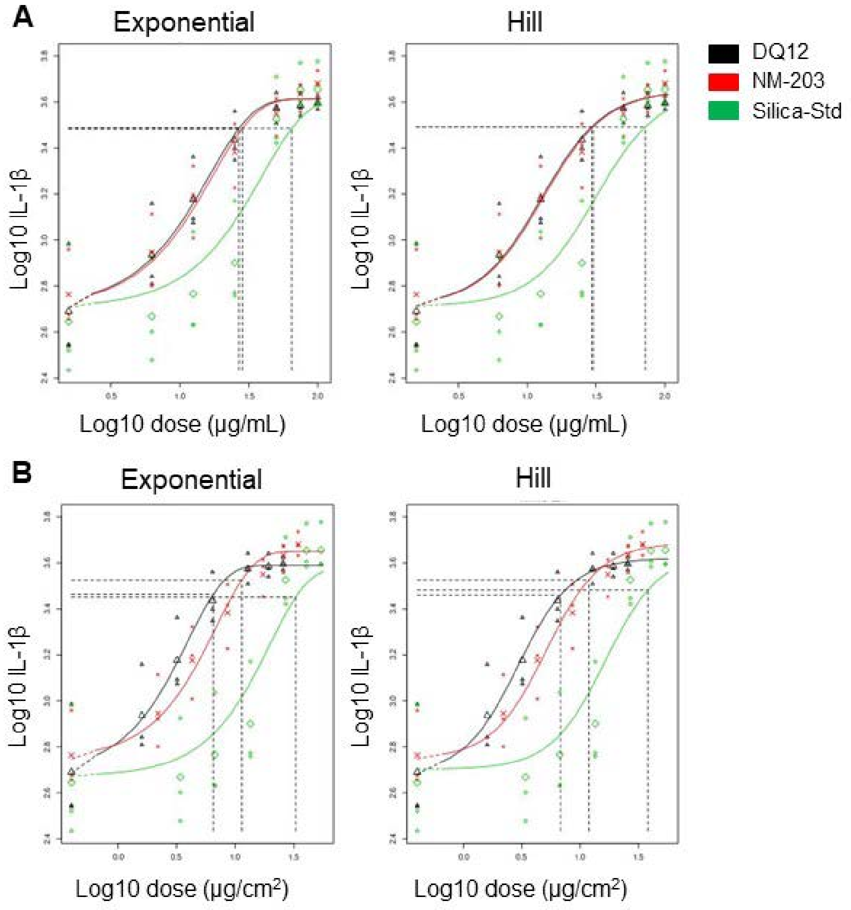

Fig. S6 PROAST dose-response curves of dTHP-1 IL-1β secretion assessed using ELISA, supporting Figure 4 in the manuscript. THP-1 cells were differentiated following the REFINE SOP. Administered doses (A) and deposited doses (B). CES = 500%. This experiment was carried out with FBS supplementation.

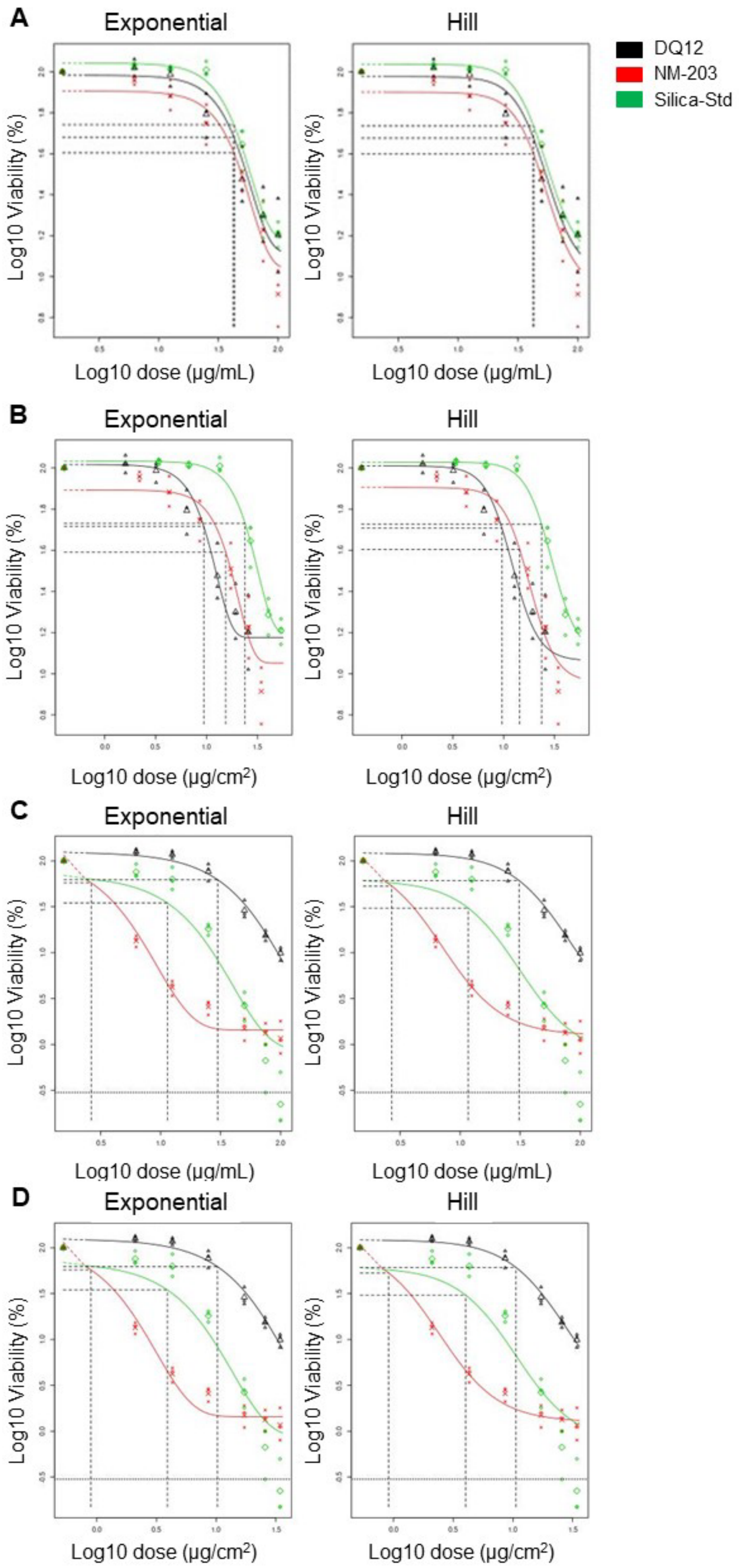

Fig. S7 PROAST dose-response curves of dTHP-1 viability with serum (A+B) and dTHP-1 viability without serum (C+D) assessed using WST-1 assay, supporting Figure 4 in the manuscript. THP-1 cells were differentiated following the REFINE SOP. Administered doses (A+C) and deposited doses (B+D). CES = 50%. This experiment was carried out with FBS supplementation.

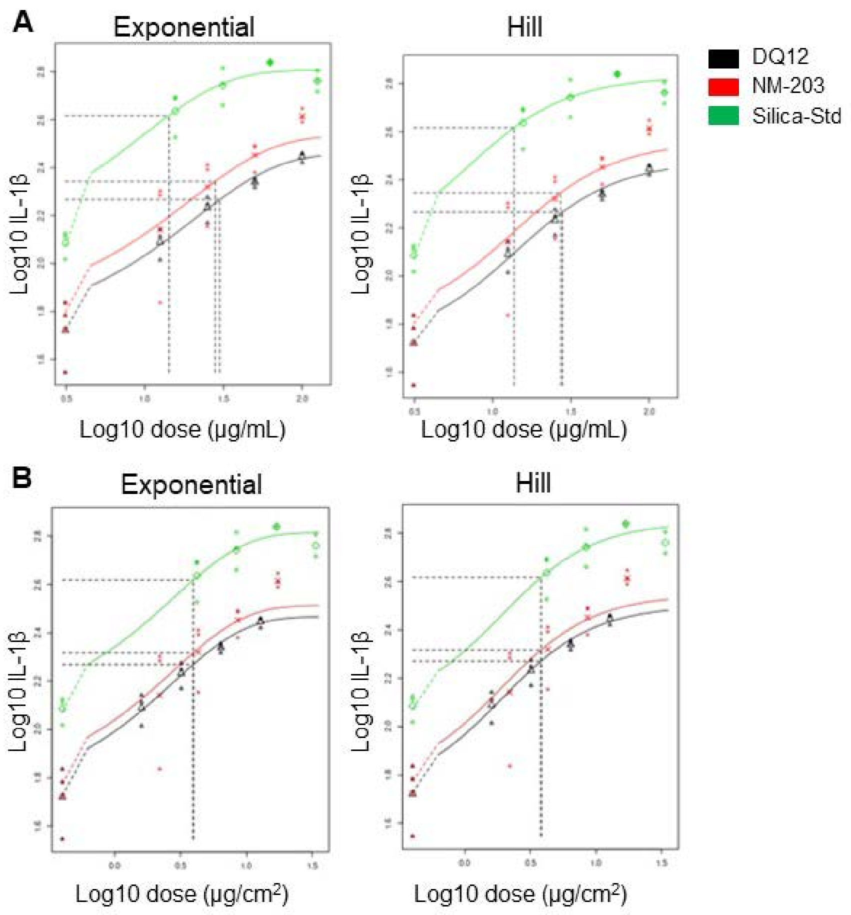

Fig. S8 PROAST dose-response curves of M0 dTHP-1 IL-1β secretion assessed using ELISA, supporting Figure 5 and 6 in the manuscript. THP-1 cells were differentiated following the Genin et al. (2015) SOP. Administered doses (A) and deposited doses (B). CES = 250%. This experiment was carried out with FBS supplementation.

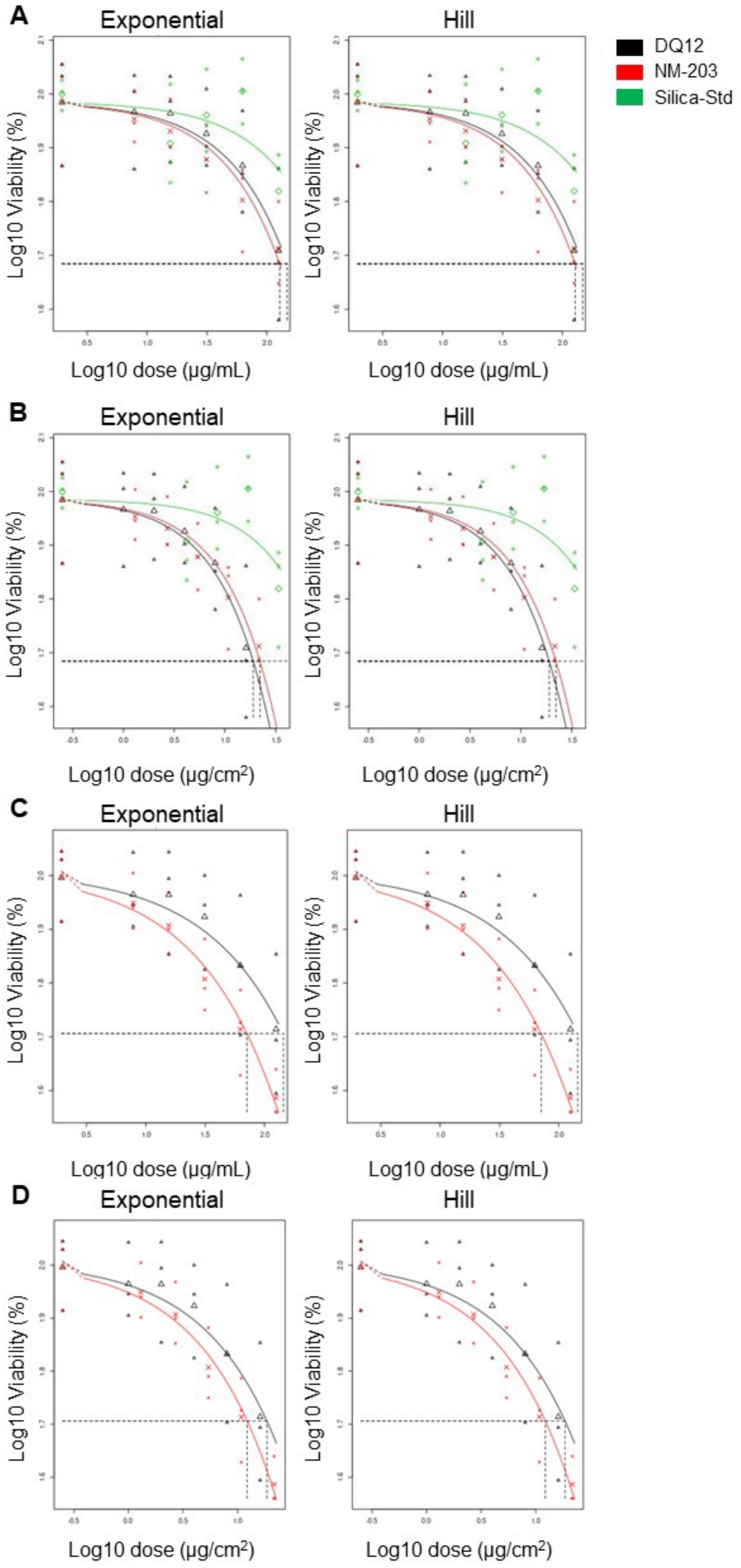

Fig. S9 PROAST dose-response curves of M0 dTHP-1 viability (A+B) and M1 dTHP-1 viability (C+D) assessed using Alamar Blue assay, supporting Figure 5 and 6 in the manuscript. THP-1 cells were differentiated following the Genin et al. (2015) SOP. Administered doses (A+C) and deposited doses (B+D). CES = 50%. This experiment was carried out with FBS supplementation.

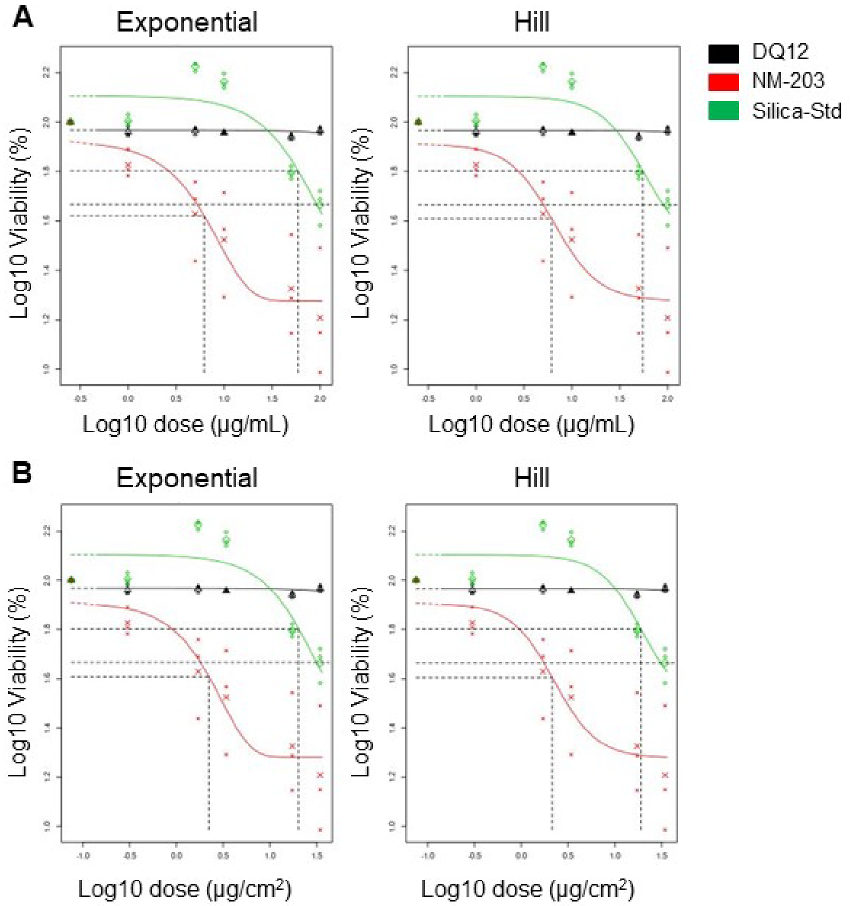

Fig. S10 PROAST dose-response curves of dTHP-1 viability assessed using MTT assay. THP-1 cells were differentiated following the same protocol as was used for ALI co-cultures. Administered doses (A) and deposited doses (B). CES = 50%. This experiment was carried out without FBS supplementation.

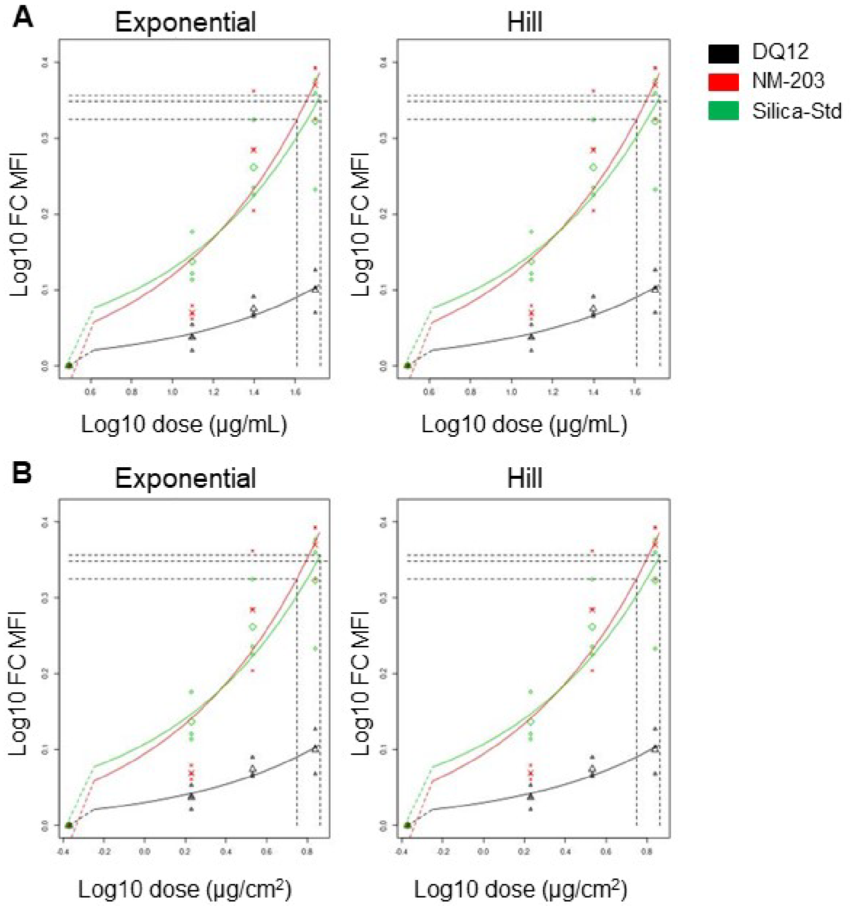

Fig. S11 PROAST dose-response curves of DCFH assay results in RAW264.7 cells, supporting Figure 7 in the manuscript. Results are expressed as fold change in median fluorescence intensity. Administered doses (A) and deposited doses (B). CES = 123%. This experiment was carried out without FBS supplementation.

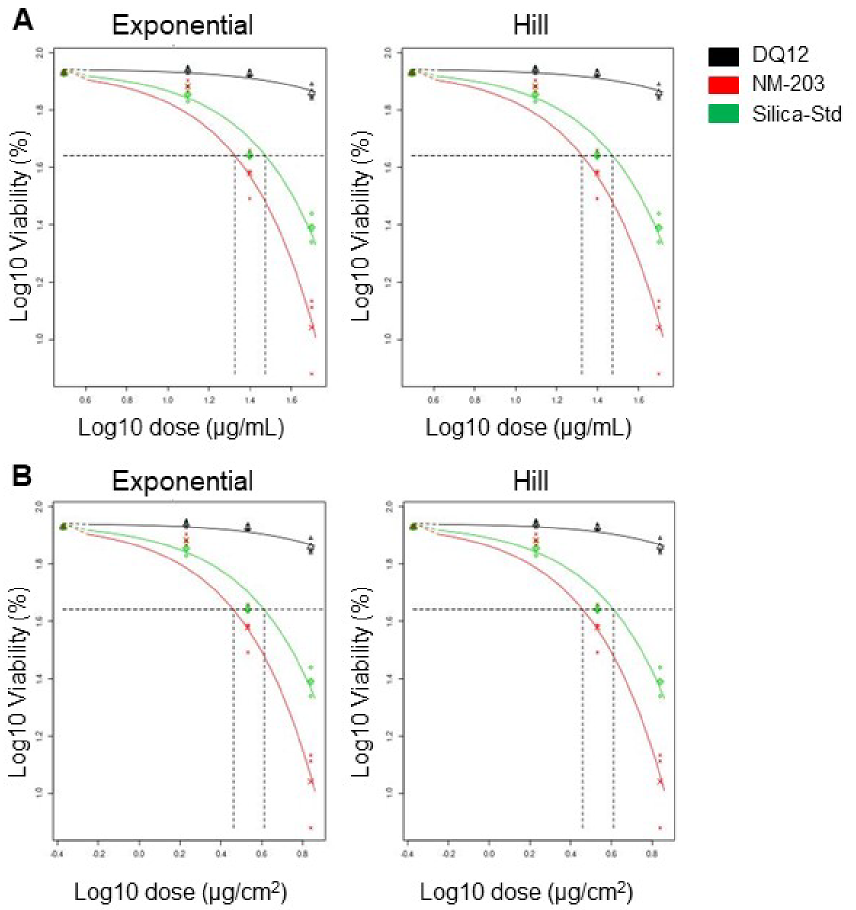

Fig. S12 PROAST dose-response curves of RAW264.7 cell viability as assessed by live-dead stain and flow cytometry, supporting Figure 7 in the manuscript. Administered doses (A) and deposited doses (B). CES = 50%. This experiment was carried out without FBS supplementation.

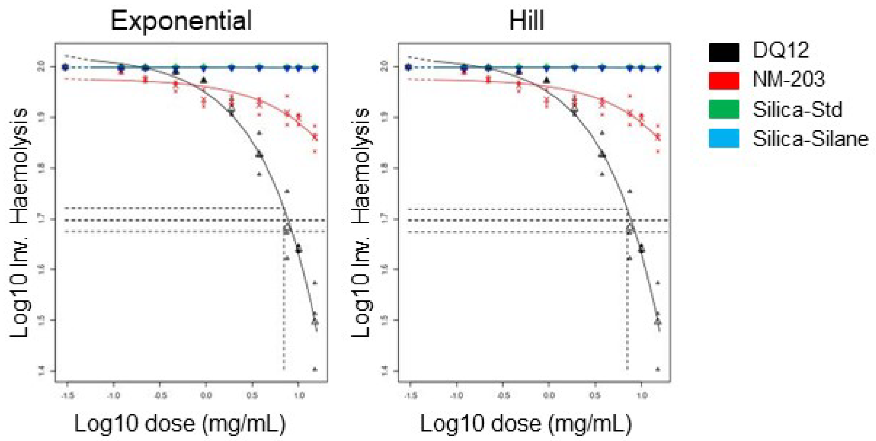

Fig. S13 PROAST dose-response curves of haemolysis assay using an inverted data-set (100% is no haemolysis, 0% is complete haemolysis), supporting Figure 8 in the manuscript. CES = 50%. Silica-Std and Silica-Silane data are from Ruijter et al. (2025).

Table S2 Detailed findings of in vivo rodent studies used for the hazard ranking.

Reference	Time points and animal strain	Particle, concentration, MMAD (µm) / GSD	PMN after exposure period (% of total lavaged cells)	PMN after recovery (% of total lavaged cells)	Histology after recovery (granuloma and fibrosis)	NOAEC/LOAEC reported
**Quartz**						
Muhle et al. (1995)	-2-year inhalation in Fisher-344 rats<br>-Recovery 6 weeks	-DQ12<br>-1 mg/m^3<br>-MMAD: 1.3 µm / 1.8	Not assessed	Not assessed	Moderate fibrosis observed for 92% of animals at 1 mg/m^3.	LOAEC: 1 mg/m^3 (only conc. tested)
Weber et al. (2024)	-90-day inhalation in unspecified rats (OECD 413 GLP)<br>-Recovery 90 or 365 days	-DQ12<br>-1 mg/m^3<br>-MMAD: 1.2 µm / 2	52.2%* (controls not specified) at 1 mg/m^3.	37.2%* (controls not specified) at 1 mg/m^3 after 90 days of recovery.	Multifocal interstitial fibrosis observed in 80% of animals after 365 days of recovery.<br>Granulomas observed after 365 days of recovery.	LOAEC: 1 mg/m^3 (only conc. tested)
Reuzel et al. (1991)	-90-day inhalation in Wistar rats<br>-Recovery 13, 26, 39, or 52 weeks	-Sikron F300<br>-60 mg/m^3<br>-MMAD not measured	48E8/L* (vs 20E8/L in controls). Values are total PMN counts in blood.	94E8/L* (vs 14 E8/L in controls) after 13 weeks of recovery, and 95 E8/L* (vs 31 E8/L in controls) after 52 weeks of recovery. Values are total PMN counts in blood.	Granuloma-like lesions observed in 10/10* animals after exposure and after 1 year of recovery.<br>Focal interstitial fibrosis observed in 10/10* animals after 1 year of recovery.	LOAEC: 60 mg/m^3 (only conc. tested)
Henderson et al. (1995)	-28-day inhalation in F-344 rats<br>-Recovery 1 week, 2 months, or 6 months	-Min-U-Sil 5<br>-0.1, 1, 10 mg/m^3<br>-MMAD: not determined for 0.1 mg/m^3; 1.3 µm / 2 (1 mg/m^3); 2.0 µm	Not elevated at 0.1 mg/m^3, 43E^3* at 1 mg/m^3, 190E^3* at 10 mg/m^3 (vs 2.6E^3 in controls).	Recovered for 1 mg/m^3, 1680E^3* for 10 mg/m^3 (vs 14.5E^3 in controls) after 6 months.	Granulomas found in 10 mg/m^3 group after 6 months of recovery. No mention of fibrosis.	NOAEC: 0.1 mg/m^3<br>LOAEC: 1 mg/m^3

		/ 2.2 (10 mg/m^3)				
Arts et al. (2007)	-5-day inhalation in Wistar rats<br>-Recovery 1 or 3 months	-Min-U-Sil 5<br>-25 mg/m^3<br>-MMAD: 2.08 µm /1.61	Not significantly elevated	Significantly elevated at 1 month and 3 months post-exposure.	Clear signs of inflammation such as alveolar inflammatory cell infiltrate and debris. The 5-day exposure period to quartz was likely too short to induce interstitial fibrosis, granulomatous lesions, and septal hypercellularity.	LOAEC: 25 mg/m^3 (only conc. tested)
**Pyrogenic silica**						
Reuzel et al. (1991)	-90-day inhalation in Wistar rats<br>-Recovery 13, 26, 39, or 52 weeks	-Aerosil 200/NM-203<br>-1, 6, 30 mg/m^3<br>-MMAD not measured	44E^8/L* (vs 20E^8/L in controls) at 30 mg/m^3. Values are total PMN counts in blood.	21E^8/L (vs 14 E^8/L in controls) after 13 weeks of recovery (30 mg/m^3 concentration). Values are total PMN counts in blood.	Focal interstitial fibrosis observed in 9/10 animals* at 30 mg/m^3. In a re-evaluation of these lung slides, the findings were diagnosed as fibrogenesis instead of fibrosis (Weber et al., 2018). The RAC later noted that not all animals and lung sections were re-evaluated and that the original tissue samples may have been damaged during the de-cover-slipping and re-staining (ECHA, 2019)<br>Granuloma-like lesions observed in 2/10 animals after 90-day inhalation and were absent after 13 weeks of recovery.	LOAEC: 1 mg/m^3
Weber et al. (2024)	-90-day inhalation in unspecified rats (OECD 413 GLP)	-SAS1**, high BET (400 m^2/g) synthetic	Increase in PMN in BALF at 2.5 (29%*) and 5 mg/m^3	Fully recovered after 90 days at all concentrations.	No fibrosis or fibrinogenesis observed. After 90 days of recovery most of the animals	NOAEC: 1 mg/m^3<br>LOAEC: 2.5 mg/m^3

	-Recovery 90 or 365 days	amorphous silica<br>-0.5, 1, 2.5, 5 mg/m^3<br>-MMAD: 2.1-3.0 µm / 2.2-3.5	(38.4%*), but not at 0.5 and 1 mg/m^3. Controls not specified.		were affected by minimal chronic interstitial inflammation. Low incidence and severity granulomas observed for all conc. and recovery groups except at 2.5 mg/m^3 after 90 days recovery.	
Arts et al. (2007)	-5-day inhalation in Wistar rats<br>-Recovery 1 or 3 months	-Cab-O-Sil M5 (BET 200 m^2/g)<br>-1, 5, 25 mg/m^3<br>-MMAD: 1.86 µm / 1.79 (1 mg/m^3); 1.94 µm / 1.70 (5 mg/m^3); 1.70 µm / 1.77 (25 mg/m^3)	Significantly elevated for 5 and 25 mg/m^3.	Back to control levels after 3 months of recovery.	Accumulation of alveolar macrophages observed after exposure at 25 mg/m^3, but not after recovery. No fibrosis or granuloma observed.	NOAEC: 1 mg/m^3<br>LOAEC: 5 mg/m^3
**Colloidal Silica**						
Anonymous (2021) and J. Arts, personal communication	-90-day inhalation in Wistar rats (OECD 413 GLP)<br>-Recovery 90 days	-CS30-236<br>-25 mg/m^3<br>-MMAD: 1.40-1.68 µm / 2.14-3.07	63.5%* of total lavaged cells (vs 4.35% in control) for males and 71.45%* (vs 1.52 % in control) for females.	8.95%* of total lavaged cells (vs 1.95% in control) for males and 3.60%* (vs 0.10% in control) for females.	Fibrosis not present. Minimal-mild (multi)focal granuloma in 19/20 animals after 90-day inhalation, and in 4/10 animals after 90 days of recovery.	LOAEC: 25 mg/m^3 (only conc. tested)
Lee and Kelly (1992)<br>Warheit et al. (1991)	-28-day inhalation in CD rats<br>-Recovery 10 days or 3 months	-Ludox CL-X (22 nm)<br>-10, 50, and 150 mg/m^3<br>-MMAD 3.7 µm / 1.9 (10 mg/m^3); 3.3 µm	0.2E^5 at 10 mg/m^3, 22E^5* at 50 mg/m^3, 45E^5* at 150 mg/m^3 for Ludox, vs 0.1E^5 in controls.	0.9E^5 at 10 mg/m^3, 0.8E^5 at 50 mg/m^3, 4E^5* at 150 mg/m^3 for Ludox, vs 0.1E^5 in controls after 3 months recovery.	Fibroblast proliferation was observed but was transient, and almost completely resolved at 50 and 150 mg/m^3. Silicotic nodular-like lesions were observed in 1/10 animals at 50 mg/m^3 and in 3/10 animals at 150 mg/m^3 after	NOAEC: 10 mg/m^3<br>LOAEC: 50 mg/m^3

		/ 2.1 (50 mg/m^3); 2.9 µm / 2.3 (150 mg/m^3)			the recovery period. Nodular macrophage aggregates were observed in 1/10 animals at 50 mg/m^3 and in 9/10 animals at 150 mg/m^3 after recovery. The observed findings were devoid of marked granulomatous inflammation.	
Landsiedel et al. (2014)	-5-day inhalation in Han rats<br>-Recovery 21 days	-SiO_2-naked (defined as Levasil 200 in (Wiemann et al., 2016))<br>-0.5, 2.5, 10, 50 mg/m^3<br>-MMAD: 1.5 µm / 3.2 (0.5 mg/m^3); 1.1 µm / 2.3 (2.5 mg/m^3); 1.4 µm / 2.3 (10 mg/m^3); 2.1 µm / 2.7 (50 mg/m^3)	Increased at 50 mg/m^3.	Increased at 10 and 50 mg/m^3.	Multifocal macrophage aggregates. Exacerbation towards a slight multifocal inflammation after recovery.	NOAEC: 2.5 mg/m^3<br>LOAEC: 10 mg/m^3
**Silane functionalized colloidal silica**						
Anonymous (2021) and J. Arts, personal communication	-90-day inhalation in Wistar rats (OECD 413 GLP)<br>-Recovery 90 days	-CC301<br>-1, 5 and 25 mg/m^3<br>-MMAD: 0.40-0.66 µm / 3.00-5.20 (1	69.48%* (vs 4.35% in control) for males and 74.85%* (vs 1.52% in control) for females at 25 mg/m^3.	14.10%* (vs 1.95% in control) for males and 13.45%* (vs 0.01% in control) for females at 25 mg/m^3.	No granuloma or fibrosis present at 1, 5 and 25 mg/m^3.	NOAEC: 1 mg/m^3 but limited findings at 5 mg/m^3<br>LOAEC: 5 mg/m^3

		mg/m^3); 0.78-1.08 µm / 3.02-4.11 (5 mg/m^3); 0.86-1.64 µm / 2.50-3.90 (25 mg/m^3)				
Landsiedel et al. (2014)	-5-day inhalation in Han rats<br>-Recovery 21 days	-SiO_2-PEG; SiO_2-phosphate; and SiO_2-amino (SiO_2-naked as core)<br>-2, 10, 50 mg/m^3<br>-MMAD: 0.8 – 1.8 µm / 2 - 4	No effects	No effects	No effects	NOAEC: 50 mg/m^3

*In all inhalation studies, animals were exposed 6 h/day, 5 days a week, unless otherwise specified. NOAEC = no observed adverse effects concentration. LOAEC = lowest observed adverse effect concentration. PMN = polymorphonuclear cells. BALF = Bronchioalveolar lavage fluid. MMAD = mass median aerodynamic diameter. PEG = polyethyleneglycol. GSD = geometric standard deviation. RAC = Committee for risk assessment (of the European Chemicals Agency, ECHA). * statistically significant elevation according to source. ** SAS2, the other pyrogenic silica particle included in this study, was not considered for this table as SAS2 was micron-sized and therefore showed much slower dissolution as compared to NM-203.*

Table S3 BMD confidence intervals and rankings for dose-response data.

Model	Particle	BMDL (µg/mL)	BMDU (µg/mL)	BMDL (µg/cm²)	BMDU (µg/cm²)	Ranking
Primary cells ALI IL-8 and MCP-1	DQ12	No effect				None
	NM-203	No effect				None
	Silica-Std	No effect				None
	Silica-Silane	No effect				None
Calu-3 + THP-1 ALI IL-8 and IL-6	DQ12	No effect				None
	NM-203	No effect				None
	Silica-Std	No effect				None
	Silica-Silane	No effect				None
Calu-3 submerged IL-8 (CES = 350%)	DQ12	2110000	Inf	797000	Inf	2
	NM-203	0.0805	22.4	0.0277	7.57	1
	Silica-Std	1630	Inf	575	Inf	2
	Silica-Silane	Not tested submerged				X
Calu-3 submerged IL-6 (CES = 3000%)	DQ12	1250	2.38E08	435	8.92E07	3
	NM-203	1.01	18.9	0.355	6.66	1
	Silica-Std	60.01	128	20.7	44.5	2
	Silica-Silane	Not tested submerged				X
dTHP-1 IL-1β, REFINE SOP (CES = 500%)	DQ12	19.6	45.2	4.6	10.9	1
	NM-203	20.4	46.4	7.76	19	1
	Silica-Std	47.3	114	22.7	60.8	2
	Silica-Silane	Not tested submerged				X
dTHP-1 IL-1β, Genin et al. (2015) SOP M0 (CES = 250%)	DQ12	15.9	44.2	2.92	5.15	1
	NM-203	13	52.6	2.92	5.15	1
	Silica-Std	7.92	30.4	2.92	5.15	1
	Silica-Silane	Not tested submerged				X
DCFH-DA (CES = 123%)	DQ12	211	675	29.5	96.7	2
	NM-203	29.6	59	4.06	8.18	1
	Silica-Std	39.9	75.1	5.47	10.4	1
	Silica-Silane	Not tested submerged				X
Haemolysis assay (inverted dataset CES = 50%)	DQ12	5900	7890	N/A	N/A	1
	NM-203	42800	73600	N/A	N/A	2
	Silica-Std	1.18E07*	1.98E08*	N/A	N/A	3
	Silica-Silane	1.08E07*	Inf*	N/A	N/A	3

BMDL = lower limit of the 95% benchmark dose confidence interval. BMDU = Upper limit of the 95% benchmark dose confidence interval. From the exponential and hill models, the lowest BMDL and the highest BMDU were taken. CES = Critical effect size. Inf = Infinite. data obtained from Ruijter et al. (2025).*

Table S4 BMD confidence intervals and rankings for dose-response data for cell viability.

Model	Particle	BMDL (µg/mL)	BMDU (µg/mL)	BMDL (µg/cm²)	BMDU (µg/cm²)	Ranking
Primary cells ALI LDH release and TEER	DQ12	No effect				
	NM-203	No effect				
	Silica-Std	No effect				
	Silica-Silane	No effect				
Calu-3 + THP-1 ALI LDH release and TEER	DQ12	No effect				
	NM-203	No effect				
	Silica-Std	No effect				
	Silica-Silane	No effect				
Calu-3 submerged MTT	DQ12	133	232	45.5	79.1	2
	NM-203	128	Inf	43.8	Inf	2
	Silica-Std	73.1	90.3	25.1	31	1
A549 submerged MTT	DQ12	295	Inf	101	Inf	3
	NM-203	142	291	48.5	99.4	2
	Silica-Std	39	45.2	13.4	15.5	1
dTHP-1 submerged MTT (same differentiation as at ALI)	DQ12	263	Inf	87.9	Inf	3
	NM-203	2.47	12.9	0.88	4.47	1
	Silica-Std	39.5	81.8	13.5	28.1	2
dTHP-1 submerged (REFINE) with serum WST-1	DQ12	36.8	46.5	7.63	11.7	1
	NM-203	36.8	46.5	11.4	18.1	1
	Silica-Std	36.8	46.5	20.7	25.9	2
dTHP-1 submerged (REFINE) w/o serum WST-1	DQ12	24.4	35.9	8.34	12.3	3
	NM-203	2.07	3.3	0.699	1.13	1
	Silica-Std	7.98	16.4	2.72	5.63	2
dTHP-1 submerged Genin et al. (2015) SOP M0 Alamar Blue	DQ12	112	227	14.4	29.2	1
	NM-203	97	176	16.8	30.6	1
	Silica-Std	191	909	51.7	247	2
dTHP-1 submerged Genin et al. (2015) SOP M1 Alamar Blue	DQ12	96.4	240	12.4	31	1
	NM-203	52.2	91.6	9.02	15.8	1
	Silica-Std	Not tested				X
BEAS-2B submerged WST-1 (same conditions as gene expression exp.)	DQ12	158	220	27.2	37.8	2
	NM-203	26.7	58.3	4.6	9.99	1
	Silica-Std	13.3	32	2.29	5.51	1
A549 submerged WST-1 (same conditions as gene expression exp.)	DQ12	440	Inf	75.5	Inf	3
	NM-203	40.4	139	6.92	23.9	2
	Silica-Std	8.35	33.2	1.43	5.71	1
RAW264.7 submerged flow cytometry	DQ12	117	209	16.4	29.5	3
	NM-203	18.3	24.1	2.49	3.29	1
	Silica-Std	27.3	32.7	3.74	4.49	2

CES = 50%. All experiments were carried out three times, with three technical replicates. Except the DQ12 BEAS-2B experiment, which was carried out two times.

Table S5 Confidence intervals and rankings of one-dose *in vitro* data.

Cell model/assay	Particle	Lower limit CI response magnitude	Upper limit CI response magnitude	Ranking
BEAS-2B submerged qPCR IL-6 (Fold change at 16 µg/mL)	DQ12	6.92	12.5	1
	NM-203	NS	NS	3
	Silica-Std	2.03*	2.86*	2
	Silica-Silane	Not tested submerged		X
A549 submerged qPCR IL-8 (Fold change at 32 µg/mL)	DQ12	NS	NS	3
	NM-203	3.28	6.51	2
	Silica-Std	20.44*	27.10*	1
	Silica-Silane	Not tested submerged		X
FRAS assay (Biological oxidative damage at 40 mg/mL)	DQ12	NS	NS	2
	NM-203	NS	NS	2
	Silica-Std	44.6	78.6	1
	Silica-Silane	50.3	57.6	1

*CI = 95% confidence interval. NS = Not significant. * = data obtained from Ruijter et al. (2025).*

References

Anonymous. (2021). *Silicon Dioxide ECHA dossier OECD TG 413 Inhalation Toxicity Study*. https://chem.echa.europa.eu/100.028.678/dossier-view/f45e0594-0618-4fda-bfd0-8108096d5ea1/90e350af-f7c9-4d22-b6c8-0c5f70074f4d_c9ab84d6-58ac-4ac8-a828-eaf539751f78?searchText=silicon%20dioxide

Arts, J. H., Muijser, H., Duistermaat, E., Junker, K., & Kuper, C. F. (2007). Five-day inhalation toxicity study of three types of synthetic amorphous silicas in Wistar rats and post-exposure evaluations for up to 3 months. *Food and chemical toxicology*, *45*(10), 1856-1867.

ECHA. (2019). Opinion proposing harmonised classification and labelling at EU level of Silanamine, 1,1,1-trimethyl-N-(trimethylsilyl)-, hydrolysis products with silica; pyrogenic, synthetic amorphous, nano, surface treated silicon dioxide. *Helsinki, Finland* (CLH-O-0000006735-67-01/F). https://echa.europa.eu/documents/10162/bf92a787-c50f-c453-7a9f-ee0446d01a91

Genin, M., Clement, F., Fattaccioli, A., Raes, M., & Michiels, C. (2015). M1 and M2 macrophages derived from THP-1 cells differentially modulate the response of cancer cells to etoposide. *BMC Cancer*, *15*(1), 577. https://doi.org/10.1186/s12885-015-1546-9

Henderson, R., Driscoll, K., Harkema, J., Lindenschmidt, R., Chang, I.-Y., Maples, K., & Barr, E. (1995). A comparison of the inflammatory response of the lung to inhaled versus instilled particles in F344 rats. *Fundamental and applied toxicology*, *24*(2), 183-197.

Landsiedel, R., Ma-Hock, L., Hofmann, T., Wiemann, M., Strauss, V., Treumann, S., Wohlleben, W., Gröters, S., Wiench, K., & van Ravenzwaay, B. (2014). Application of short-term inhalation studies to assess the inhalation toxicity of nanomaterials. *Particle and Fibre Toxicology*, *11*, 1-26.

Lee, K. P., & Kelly, D. P. (1992). The Pulmonary Response and Clearance of Ludox Colloidal Silica after a 4-Week Inhalation Exposure in Rats. *Toxicological Sciences*, *19*(3), 399-410. https://doi.org/10.1093/toxsci/19.3.399

Muhle, H., Kittel, B., Ernst, H., Mohr, U., & Mermelstein, R. (1995). Neoplastic lung lesions in rat after chronic exposure to crystalline silica. *Scandinavian journal of work, environment & health*, 27-29.

Reuzel, P. G., Bruijntjes, J. P., Feron, V. J., & Woutersen, R. A. (1991). Subchronic inhalation toxicity of amorphous silicas and quartz dust in rats. *Food Chem Toxicol*, *29*(5), 341-354. https://doi.org/10.1016/0278-6915(91)90205-l

Ruijter, N., Zanoni, I., Persson, D., Arts, J., Carriere, M., Guiot, A., Persson, M., Katsumiti, A., Marshall, J., Boyles, M., Cassee, F. R., & Braakhuis, H. M. (2025). Hazard screening of colloidal silica nanomaterials with varying degrees of silane surface functionalization: a safe-by-design case study *Particle and Fibre Toxicology*(In press). https://doi.org/https://doi.org/10.1186/s12989-025-00629-6

Warheit, D. B., Carakostas, M. C., Kelly, D. P., & Hartsky, M. A. (1991). Four-week inhalation toxicity study with Ludox colloidal silica in rats: pulmonary cellular responses. *Fundamental and applied toxicology*, *16*(3), 590-601.

Weber, K., Bosch, A., Bühler, M., Gopinath, C., Hardisty, J. F., Krueger, N., McConnell, E. E., & Oberdörster, G. (2018). Aerosols of synthetic amorphous silica do not induce fibrosis in lungs after inhalation: Pathology working group review of histopathological specimens from a subchronic 13-week inhalation toxicity study in rats. *Toxicology Research and Application*, *2*, 2397847318805273. https://doi.org/10.1177/2397847318805273

Weber, K., Bruer, G., Krueger, N., Schuster, T. B., Creutzenberg, O., & Schaudien, D. (2024). Regenerative and progressing lesions in lungs and lung-associated lymph nodes from fourteen 90-day inhalation studies with chemically different particulate materials. *Toxicology Letters*, *399*, 49-72. https://doi.org/https://doi.org/10.1016/j.toxlet.2023.12.011

Wiemann, M., Vennemann, A., Sauer, U. G., Wiench, K., Ma-Hock, L., & Landsiedel, R. (2016). An in vitro alveolar macrophage assay for predicting the short-term inhalation toxicity of nanomaterials. *Journal of Nanobiotechnology*, *14*, 1-27.
